# Truncated HDAC9 identified by integrated genome-wide screen as the key modulator for paclitaxel resistance in triple-negative breast cancer

**DOI:** 10.7150/thno.44997

**Published:** 2020-09-02

**Authors:** Bi Lian, Yu-Chen Pei, Yi-Zhou Jiang, Meng-Zhu Xue, Da-Qiang Li, Xiao-Guang Li, Yi-Zi Zheng, Xi-Yu Liu, Feng Qiao, Wei-Li Sun, Hong Ling, Min He, Ling Yao, Xin Hu, Zhi-Ming Shao

**Affiliations:** 1 Department of Breast Surgery, Key Laboratory of Breast Cancer in Shanghai, Fudan University Shanghai Cancer Center, Fudan University, Shanghai, China.; 2 Department of Oncology, Shanghai Medical College, Fudan University, Shanghai, China.; 3 Precision Cancer Medicine Center, Fudan University Shanghai Cancer Center, Shanghai, China.; 4 Laboratory of Systems Biology, Shanghai Advanced Research Institute, Chinese Academy of Sciences, 200031 Shanghai, China.

**Keywords:** breast cancer, paclitaxel resistance, CRISPR screen, HDAC9, MITR

## Abstract

**Rationale:** Paclitaxel resistance is a major concern when treating triple-negative breast cancer (TNBC) patients. We aimed to identify candidates causing paclitaxel resistance and explore their significance in TNBC therapeutics.

**Methods:** A genome-wide CRISPR screening, integrated with transcriptome analyses, was performed to identify candidates involved in paclitaxel-resistant TNBCs. Cell proliferation, cytotoxicity, immunofluorescent staining, and xenograft assays were conducted to verify the phenotypes of paclitaxel resistance induced by candidate genes, both* in vitro* and* in vivo*. RNA sequencing, Western blotting, and chromatin immunoprecipitation assays were used to explore the underlying mechanisms.

**Results:** MEF2-interacting transcriptional repressor (MITR), the truncated isoform of histone deacetylase 9 (HDAC9) lacking the deacetylation domain, was enriched in paclitaxel-resistant cells. Elevated MITR expression resulted in increased interleukin-11 (IL11) expression and activation of downstream JAK/STAT3 signaling. Mechanistically, MITR counteracted MEF2A-induced transcriptional suppression of IL11, ultimately causing paclitaxel resistance. By contrast, pharmacological inhibition of JAK1/2 by ruxolitinib reversed paclitaxel resistance both *in vitro* and *in vivo*.

**Conclusion:** Our *in vitro* and *in vivo* genetic and cellular analyses elucidated the pivotal role of MITR/MEF2A/IL11 axis in paclitaxel resistance and provided a novel therapeutic strategy for TNBC patients to overcome poor chemotherapy responses.

## Introduction

Triple-negative breast cancer (TNBC) is the breast cancer subtype with the poorest prognosis, where chemotherapy is the first-line treatment option 1. Taxanes, a group of microtubule inhibitors, shows significant anti-tumor effects when treating patients with both early and metastatic TNBC. One of the first generation taxanes, paclitaxel, is currently the most commonly-used agent for TNBC treatment 2. However, its efficacy is greatly limited by primary or acquired drug resistance 3. One common drug resistance target, reported in various cancer types, is the adenosine triphosphate (ATP)-binding cassette (ABC) transporter family 4. Other mechanisms relevant to paclitaxel resistance include tubulin alteration, cell survival, and drug metabolism 5. Despite decades of efforts, there is a lack of therapeutic targets to overcome paclitaxel resistance.

The high-throughput CRISPR-Cas9 gene knockout screening library provides an effective method to functionalize candidate targets for modulating drug resistance 6. We combined the genome-wide single guide RNA (sgRNA) library screening and RNA sequencing to identify genes driving paclitaxel resistance. Furthermore, the relapse-free survival of TNBC patients after chemotherapy was examined. Overall, 8 candidates were validated, including histone deacetylase 9 (HDAC9).

The HDAC family, consisting of classes I, IIa, IIb and IV proteins, determines the acetylation status of histones 7. HDACs repress gene transcription by deacetylating histones, leading to chromatin compaction 8. Class I HDACs, in particular, are epigenetically involved in oncogenesis. As a result, inhibitors for this class, yielding accumulation of acetylated histones, are able to inhibit tumor progression 9. However, the role of class IIa HDACs with weak deacetylation activity, such as HDAC9, in cancer remains elusive.

Class IIa HDACs, uniquely among HDAC classes, come in both full-length and truncated isoforms, the latter lacking the conserved deacetylase domain 10. These truncated isoforms were first discovered as the myocyte-specific enhancer factor 2 (MEF2)-interacting transcription repressor (MITR), which binds to the N-terminal MEF2 domain 11. The HDAC-MEF complex has been reported to play pivotal roles in some biological functions, such as connecting neuronal activity to the muscle 12, controlling the pancreatic endocrine products 13, and promoting tumorigenesis of lymphomas 14.

In this study, we systematically explored the paclitaxel-resistant candidates by combining the transcription analyses of paclitaxel-resistant TNBC cells, readouts of a genome-wide CRISPR screen, and clinical prognoses associated with gene expression. We identified the pivotal role of the truncated HDAC9 isoform (MITR) in modulating paclitaxel resistance in TNBC tumors, via its downstream IL11/JAK/STAT3 axis. Thus, our study provided important insights into JAK1/2 inhibition that could be an attractive strategy to reverse MITR-induced paclitaxel resistance for TNBC patients.

## Materials and Methods

### Cell culture

MDA-MB-231, MDA-MB-549, MDA-MB-436, MDA-MB-468 and HS-578T cell lines of human breast cancer were obtained from the Shanghai Cell Bank Type Culture Collection Committee (CBTCCC, Shanghai, China). The cell lines were cultured in mycoplasma-free complete growth medium as recommended by the manufacturer. The culture strategy for the paclitaxel-resistant MDA-MB-231 cell line was previously described 15. All cell lines were identified by CBTCCC using DNA profiling and evaluated for cell line quality by HD Biosciences every 3 months. They were then cultured in L-15 medium (Gibco, USA) with 10% fetal bovine serum (Gibco, USA), and placed in a 5% CO_2_, 37 °C cell culture incubator (Thermo Scientific, USA). The cells used in the experiments were passaged fewer than six times.

### RNA sequencing and data analysis

RNA was extracted from paclitaxel-resistant and wild type MDA-MB-231 cell lines using mirVana miRNA Isolation Kit (Thermo Fisher, USA), and then prepared using TruSeq Stranded mRNA LTSample Prep Kit (Illumina, USA). The HiSeq-2500 platform (Illumina, USA) was used for sequencing. Low-quality raw data were removed using NGS QC Toolkit to obtain clean reads. These clean reads were mapped to the reference genome hg38 using hisat2, and calculated using cufflinks to obtain TPM (Transcripts per Kilobase of exon model per Million mapped reads) values for each gene. Differentially expressed genes (DEGs) were identified using the DESeq package, and a fold change ≥ 2 or ≤ 0.5 was set as the threshold.

### Genome-wide CRISPR Screen and data processing

The genome-wide CRISPR knockout (GeCKO v2) library was purchased from Addgene and was expanded to 1000× using an electronic transfection method. MDA-MB-231 cells were transduced with the pooled lentiviral library (GeCKO v2 library) at a low multiplicity of infection (MOI) value of 0.3. To ensure both the efficiency and the coverage of infection, a large-scale spin-infection of 1.5 × 10^8^ cells was adopted in 12-well plates (Falcon, USA), with 1.5 × 10^6^ cells per well. After 2 h of high-speed centrifugation of each plate at 2000 rpm, the infection was complete, and the cells were moved into larger flasks (Falcon, USA). After 7 d of puromycin (Invitrogen, USA) selection, the surviving cells were considered to be the day 0 sample, and 3 × 10^7^ of these cells were stored for further processing. The remaining cells continued to be cultured for paclitaxel resistance screening, with 3 × 10^7^ cells per sample. After culturing, 3 × 10^7^ cells were collected from each sample, and were subjected to DNA extraction using a Blood & Cell Culture Midi kit (Qiagen, USA). Nested PCR was then performed on the extracted DNA, followed with Herculase Ⅱ Fusion DNA Polymerase (Agilent, USA) to construct the next-generation sequencing library. The design of the PCR primers used has been previously reported, and the cycle numbers of the two steps for each sample were determined using a qPCR curve, lying in the exponential growth period. The Hiseq-2500 platform (Illumina, USA) was used for sequencing, and each sample was sequenced to produce nearly ∼40 million reads, in order to achieve 300 × coverage over the CRISPR library. Raw data were demultiplexed using the FASTX-Toolkit to discard low-quality reads and to assemble reads into each sample according to different barcodes. Subsequently, the sgRNA reads were aligned to the GeCKO v2 library reference using Bowtie aligner and normalized. Further analysis of the gene expression was performed with MAGeCK 16.

### Plasmid construction and cell transfection

sgRNA sequences of target genes were selected from the GeCKO library. To construct the sgRNA plasmids, we synthesized sgRNA oligoes and ligated two annealed oligoes into the BsmBI-digested lentiGuide-Puro plasmids (Addgene 52963). Detailed information is provided on the Sanjana Lab website. The expression plasmids for HA-FLAG-MITR, HA-FLAG-HDAC9a and HA-FLAG-MEF2A were constructed by Gateway technology (Invitrogen, USA). The entry vector was pDONR™223 (Invitrogen, USA), and the destination vector was pDEST™-HA-FLAG (Invitrogen, USA). The shRNA sequences of shMITR, shHDAC9a-1, and shHDAC9a-2 were 5'- AAAGATTTAGCTCCAGGAT-3', 5'- GAGGAAATACAGCTTGTTCAT -3' and 5'- AGGCCTTGGAGAAGGGTACAA -3', and the shRNA plasmids were supplied by Gene Chem Inc. (China).

We used TOP10 competent cells (TaKaRa, Japan) for transfection with plasmids, selected monoclonal colonies on a resistance plate, and performed Sanger sequencing (Sangon Biotech, China) to ensure the correct plasmids were obtained.

### Virus packaging and infection

We used HEK293T cells and Lipo2000 transfection reagent for virus packaging. For lentivirus packaging, lentiCas9-Blast (Addgene 52962), sgRNA and shRNA plasmids were mixed with pMD2G and psPAX2 at a ratio of 4:1:2. For retrovirus packaging, cells were co-transfected with expression plasmids containing HA and FLAG tags at the N-terminus, VSVG, and gag-pol at a ratio of 2:1:1. After 48 h, viral supernatants were collected, and infected cells were treated with 8 mg/mL Polybrene (Sigma, USA). After a further 48 h of infection, cells were subjected to 2 mg/mL puromycin (Sigma, USA) for 5 d, with the exception of cells infected with virus expressing Cas9, which were instead selected with 10 mg/mL blasticidin (Sigma, USA). To generate the sgRNA knockout stable cell line, wide type cells were initially infected with virus expressing Cas9. Afterwards, viruses carrying sgRNA were introduced into Cas9-expressing cells.

### RNA isolation and RT-qPCR

RNA was isolated using TRIzol reagent (Invitrogen, USA) and was reverse transcribed to cDNA using a PrimeScript RT Reagent Kit with gDNA Eraser (TaKaRa, Japan). These experiments were performed according to the manufacturer's recommended protocols. Real-time PCR was carried out on an ABI Prism 7300 detection system using SYBR premix Ex Taq (TaKaRa, Japan), and the ΔΔCt method was used to comparatively quantify the mRNA levels. U6 gene expression served as the internal control. The primer sequences used to detect mRNA levels are listed in **Table S1**. All samples were run in triplicate.

### Protein extraction and Western blotting

Protein was extracted from different cell samples using T-PER Tissue Protein Extraction Reagent (Thermo Fisher Scientific, USA), supplemented with protease and phosphatase inhibitors (Solarbio, China). After determining the protein concentration, equal amounts of protein lysates of different samples were separated via SDS-PAGE and were transferred to PVDF membranes (Millipore, USA). The membranes were blocked for 60 min with 5% BSA in TBS-T and blotted with primary antibodies for 12-16 h at 4 ℃. The following antibodies were used: Mouse monoclonal anti-GAPDH (Proteintech, 60004-1-Ig, 1:5000), rabbit monoclonal anti-FLAG (Cell Signaling, 14793, 1:1000), mouse monoclonal anti-α-tubulin (Cell Signaling, 3873, 1:5000), rabbit polyclonal anti-MITR (Abcam, ab59718, 1:1000), rabbit polyclonal anti-HDAC9a (Sigma, SAB4503694, 1:500 ), rabbit polyclonal anti-HDAC9 (Thermo Fisher, PA5-11246, 1:500), rabbit monoclonal anti-acetyl-α-tubulin (Lys40) (Cell Signaling, 5335, 1:1000), rabbit polyclonal anti-cyclin B1 (Cell Signaling, 4138, 1:1000), rabbit polyclonal anti-MEF2A (Abcam, ab86755, 1:1000), rabbit polyclonal anti-STAT3 (Cell Signaling, 4904, 1:1000) and rabbit monoclonal anti-Phospho-STAT3 (Tyr705) (Cell Signaling, 9145, 1:1000). Subsequently, the membranes were incubated with HRP-conjugated goat anti-rabbit or goat anti-mouse antibody (Jackson Immuno Research; 1:5,000). An ECL western blotting detection reagent (Millipore, USA) was used to detect signals. Images were acquired with an Amersham Imager 600 (GE Healthcare, USA).

### Cytotoxicity assay

The MDA-MB-231 and MDA-MB-549 cells were seeded into a 96-well plate at 2.5×10^3^ cells per well. The already existing medium was replaced in the following day with medium containing serially diluted paclitaxel or ruxolitinib. After 72 h, the medium was withdrawn, and a medium containing Cell Counting Kit-8 (CCK-8) assay reagent (Dojindo, Japan) was added. After 4 h, the absorbance of each well was obtained using a microplate reader at 450 nm. GraphPad Prism 7 software was used to obtain the maximal inhibitory concentration (IC_50_).

### Immunofluorescent staining

Cells were plated on glass coverslips in a 12-well plate and was treated with 10 nM paclitaxel, or the same amount of DMSO, for 12 h after cell adherence. All groups were run in triplicate. The cells in each well were fixed with 4% paraformaldehyde (Sigma, USA) for 20 min, permeabilized in 0.3% Triton X-100 (Thermo Fisher, USA) for 10 min, blocked with 5% serum for 1-2 h, and then probed overnight at 4 ℃ with mouse monoclonal anti-α-tubulin antibody (Cell Signaling, 3873, 1:500). Next, the coverslips were washed, and the cells incubated with Alexa 488-conjugated goat anti-mouse secondary antibody (Thermo Fisher, A32723, 1:500) for 1 h at room temperature. Nuclei were then labeled with DAPI (Thermo Fisher, USA), and slides of different groups were observed with a confocal microscope (Leica, Germany).

### Cell cycle analysis

The HDAC9-knockout and control MDA-MB-231 and BT-549 cells were treated with 10 nM paclitaxel or DMSO for 16 h. The cells were then fixed in ice-cold 70% ethanol (Thermo Fisher, USA) overnight and stained with PI/RNase Staining Buffer (BD Biosciences, USA) for 30 min in the dark. At least 20,000 cells from each sample were examined with a Cytomics FC 500 flow cytometer (Beckman Coulter, USA) and analyzed with CXP software (Beckman Coulter, USA).

### RNA Interference

siRNA oligoes to silence the indicated genes were designed and purchased from RiboBio Inc. (China). The target sequences were as follows:

- siControl: 5′-GCGACCAACGCCUUGAUUG-3′;
- siIL11-1: 5′- UCAGUUCACAGUCCACGUC -3′;
- siIL11-1: 5′- UCAGAAGUCGUCGUCGUCA -3′;
- siMEF2A-1: 5′- AAUUGCAACUCGACCGACG -3′;
- siMEF2A-2: 5′- AAGUAAGGUUCUAAUGGUG -3′.

### Chromatin immunoprecipitation

Chromatin immunoprecipitation (ChIP) experiments were performed mostly according to the manufacturer's instructions (EZ-ChIP™ kits, Millipore, USA). Briefly, for each ChIP assay, 5 × 10^6^ cells expressing MITR-FLAG were employed. Chromatin was cross-linked with 1% formaldehyde (Sigma, USA) and sheared using a Bioruptor UCD-200 (Diagenode, Belgium), with pulses of 30 s on and 30 s off for 30 min. The samples were then immunoprecipitated overnight with rabbit monoclonal anti-FLAG (Cell Signaling, 14793, 10 μg), or the same amount of control antibodies (Cell Signaling, 10 μg). The next day, the cross-links in the protein-antibody-DNA complexes were removed by proteinase K to obtain the FLAG-associated and negative control DNA samples. For the ChIP-qPCR assay, the two DNA samples were used as the DNA template, and primers targeting different regions of the IL11 promoter were designed (**Table S1**).

### Immunoprecipitation

For the immunoprecipitation experiments, cell lysates were prepared in IP buffer (1% Nonidet P-40, 150 mM NaCl, 50 mM Tris-HCl (pH = 7.4), 10 mM NaF, 1 mM sodium orthovanadate, 10 mM N-ethyl-amide with 1 × protease inhibitors (Complete protease inhibitor cocktail; Roche, Lewes, United Kingdom) and precleared lysate was immunoprecipitated with anti-MITR antibodies (ABCAM ab59718) overnight and protein A/G-sepharose for 2 h. The sepharose beads were then washed in lysis buffer and boiled to obtain the protein lysates. The subsequent steps were as described in the section on Western Blotting.

### Immunohistochemistry

The TMA sections were retrieved from 39 cases of breast cancer diagnosed between the years 2004 to 2007 with clinical follow-up data and were generated by the Department of Pathology at Fudan University Shanghai Cancer Center. The clinicopathologic characteristics of the studied cohort are summarized in **Table S2**. The TMA was composed of duplicate cores from different areas of the same tumor and were incubated overnight with the primary antibodies including rabbit polyclonal anti-MITR (ABCAM; 1:100, catalog no. ab59718) and rabbit polyclonal anti-MEF2A (ABCAM; 1:100, catalog no. ab86755). For each antibody, TMAs representing duplicate samples from each case were stained and scored semi quantitatively. The intensity of staining was scored as follows: 1 = weak, 2 = moderate, 3 = strong. The percentage of cells positively stained was scored as follows: 1 ≤ 25%, 2 ≤ 50%, 3 ≤ 75%, 4 > 75%. For each case, a final score was obtained by multiplying the score of intensity by the score of percentage. For clinical analysis, disease-free survival (DFS) was defined as the time from the date of primary surgery to the date of relapse/breast cancer-specific death or 2014.10.31. Patients with a study end date or loss of follow-up were censored. The analysis of DFS and OS was derived from a Kaplan-Meier estimate and compared using the log-rank test. The correlation analyses were performed using a Pearson c2 test.

### Animal models

All animal experiments were approved by the Fudan Animal Ethics committee (approval number, 2017-031). All experiments were carried out according to the Guide for the Care and Use of Laboratory Animals (NIH, 8th Edition, 2011). We used Female BALB/c nude mice (6-9-week old, weight 15-16 g) for all animal experiments. All mice were bred and cared for according to the AAALAC standard in Fudan University's specific-pathogen-free facility. For each mouse, 5 × 10^6^ MDA-MB-231 cells were subcutaneously injected into the right flank, and tumor growth was measured every 4 d until the average tumor volume reached 100 mm^3^. For HDAC9 knockout assays, 12 mice were assigned into 2 groups and were injected by HDAC9 knockout cells and control cells. Each group was given an intraperitoneal injection of 10 ng/kg paclitaxel or NS every 3 d. For the combined paclitaxel and ruxolitinib assay, 30 mice were assigned into 2 groups and were injected by MITR knockdown cells and control cells. Each group was divided into 3 groups, which were NS only, paclitaxel combined with NS (PTX+NS) and paclitaxel combined with ruxolitinib (PTX+RUX). The paclitaxel dose was 5 ng/kg, while it was 100 ng/kg for ruxolitinib. Tumor volumes and mouse weights were measured every 3 d. 3 d after the last drug injection, CO_2_ inhalation was used to euthanize the mice, followed by removal of the tumors and their storage in liquid nitrogen.

### Kaplan-Meier Plotter analysis

To select target genes that exhibited significance in TNBC patient prognosis, we used Kaplan-Meier Plotter, a public clinical database, to assess the probe datasets of 6,234 breast cancer patients with the following restrictions: 1) TNBC breast cancer, 2) Systemically treated patients, 3) Auto-select best cut off. The analysis method is described on the website (http://kmplot.com/analysis/index.php?p=background).

### TNBC data analysis

We used RNA sequencing information of 80 TNBC patients from the Fudan University Shanghai Cancer Center (FUSCC) for quantifying genes at the transcript level. Kallisto software 17 was used for quantifying transcripts, with human genome reference version GRCh37/hg19.

### GEO data analysis

We searched for GEO datasets, representing TNBC patients who received neoadjuvant chemotherapy, including paclitaxel, and evaluated differences in HDAC9 and IL11 expression in pCR (complete pathological remission) and RD (residual disease) cohorts using R package. The data included in this manuscript are in agreement with the GEO publication guidelines.

### Statistical analysis

All data are expressed as mean ± sd. To compare two groups of data, two-sided Student's t-test was utilized. The random number table method was used to randomly assign mice into different groups. Statistical analysis was performed with GraphPad Prism 7 and R software. All experiments were repeated at least three times. Differences were considered statistically significant at *p* < 0.05.

## Results

### Genome-wide CRISPR/Cas9 screen integrated with transcriptome analyses for the identification of paclitaxel-resistant candidates

**Figure 1:**
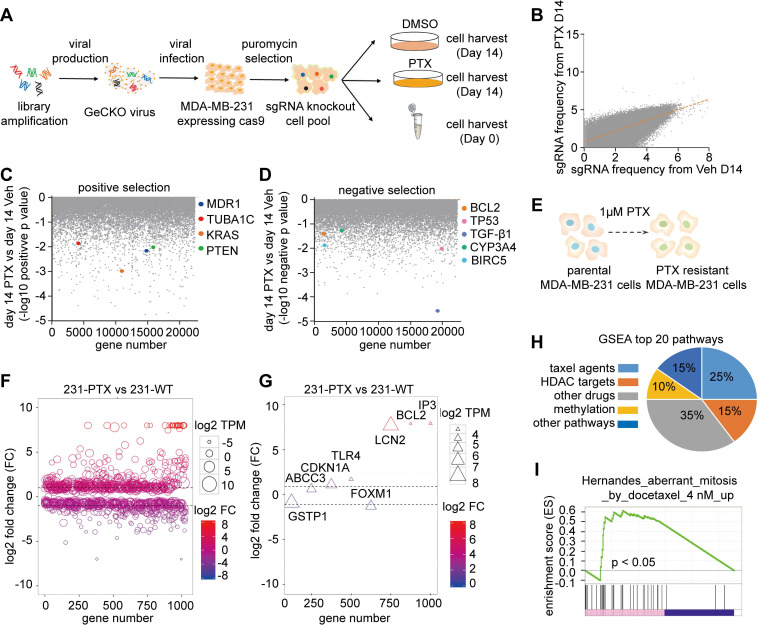
** Integrated analyses of genome-Wide CRISPR/Cas9 screen and transcriptome sequencing.** (A) Workflow of genome wide CRISPR/Cas9 knockout screening with PTX treatment. (B) Single guide RNA (sgRNA) read variations on day 14 after PTX treatment, compared to DMSO treatment. (C) Genes known to sensitize cellular response to PTX treatment. (D) Well-known resistant genes in response to PTX treatment. (E) Diagram illustrates construction of paclitaxel-resistant MDA-MB-231 cells. The cells were treated with 1 µM paclitaxel for 24 h, then changed to normal culture medium for 2 weeks. This procedure was repeated 12 times. (F) Bubble chart exhibiting significant differentially expressed genes with a log_2_-fold change (FC) ≤ -1 or ≥ 1. Bubble size represents the value of log_2_ TPM in 231-PTX cells. (G) Triangle chart confirmed previously-reported genes playing a critical role in paclitaxel resistance in cancer. The size of the triangle represents the value of log_2_ TPM in 231-PTX cells. (H) Distribution of the top 20 GSEA drug resistant-associated pathways: Taxol agent-related pathways constitute 25% of the total pathways. (I) One of the GSEA enrichment analyses among the top 20 Taxol-related pathways.

An *in vitro* genome-wide CRISPR/Cas9 knockout pooled screen was performed to identify genes involved in paclitaxel resistance in one of the TNBC cell lines, MDA-MB-231. The GeCKO v2 sgRNA library, based on the dual-vector lentiviral GeCKO system and containing 123,411 unique sgRNAs, targeting 19,050 human genes, was used. A lentiviral pooled sgRNA virus was then constructed and infected with MDA-MB-231 cells to express Cas9 at a low MOI, ensuring that each cell carries one unique sgRNA from the aforementioned library. Following puromycin selection, the cells were treated with paclitaxel (1 M) for 2 weeks. This concentration was determined using the cell proliferation assay (**Figure S1A**). The day 0 sample was collected as the baseline (Base). Additionally, day 14 (D14) sample after paclitaxel (PTX) or DMSO (Veh) treatments with two replicates were collected (**Figure 1A**). Subsequently, sgRNA regions in different samples were amplified from the genomic DNA and subjected to next-generation sequencing (NGS) for quantification. After normalization of the quantification results, some sgRNAs were found to dramatically vary between PTX-treated and Veh control samples at day 14, suggesting that they may play important roles in PTX resistance (**Figure 1B**). Different groups of datasets exhibited dispersive sgRNA counts from Day 0 to Day 14 samples (**Figure S1B**) and high concordance within replicates (**Figure S1C**). Next, we applied MAGeCK analysis to obtain gene rankings, according to sgRNA representations, and compared the PTX group to the Veh group at the same time points. This statistical algorithm defined negative selection as genes with significantly depleted sgRNAs, and positive selection as genes with significantly enriched sgRNAs under PTX treatment. Known paclitaxel-sensitive genes MDR1 18, TUBA1C 19, KRAS 20 and PTEN 21, plus known paclitaxel-resistance genes BCL2 22, TP53 23, TGF-β1 24, CYP3A4 25, BIRC5 26 and RRM2B 27; exhibited remarkable changes (*p* < 0.05) in both positive (**Figure 1C**) or negative selections (**Figure 1D**).

We also explored transcriptional features of paclitaxel-resistant mammary cancer cells. We established that in paclitaxel-resistant MDA-MB-231 (231-PTX) cells, the half-maximal inhibitory concentration (IC_50_) value in response to paclitaxel administration was nearly 15-fold higher than that of their parental MDA-MB-231 (231-WT) cells (**Figure 1E**). To investigate the candidates involved in paclitaxel resistance, RNA sequencing (RNA-Seq) was conducted to obtain transcriptome profiles of 231-PTX and 231-WT cells (**Figure S1D**). The transcriptional features were then analyzed, in which fold changes ≤ 1/2 or ≥ 2 were defined as significant (**Figure 1F**). Some previously reported paclitaxel-resistant genes, such as BCL2A1 28, ABCC3 29, CDKN1A 30, and TLR4 31, exhibited significantly different expression in our experiments (**Figure 1G**). All drug resistance-related pathways were also explored by the gene set enrichment analysis (GSEA) (**Table S3**). The results indicated that among the top 20 pathways (*p* < 0.05), those related to taxol agents (*e.g.* paclitaxel, docetaxel) ranked first; involving 25% of all pathways (**Figure 1H**). One of the top 20 taxol-related pathways was aberrant mitosis (**Figure 1I**). We also analyzed the top 20 KEGG pathways, which included ABC transporters involved in multiple drug resistance (**Figure S1E**).

**Figure 2:**
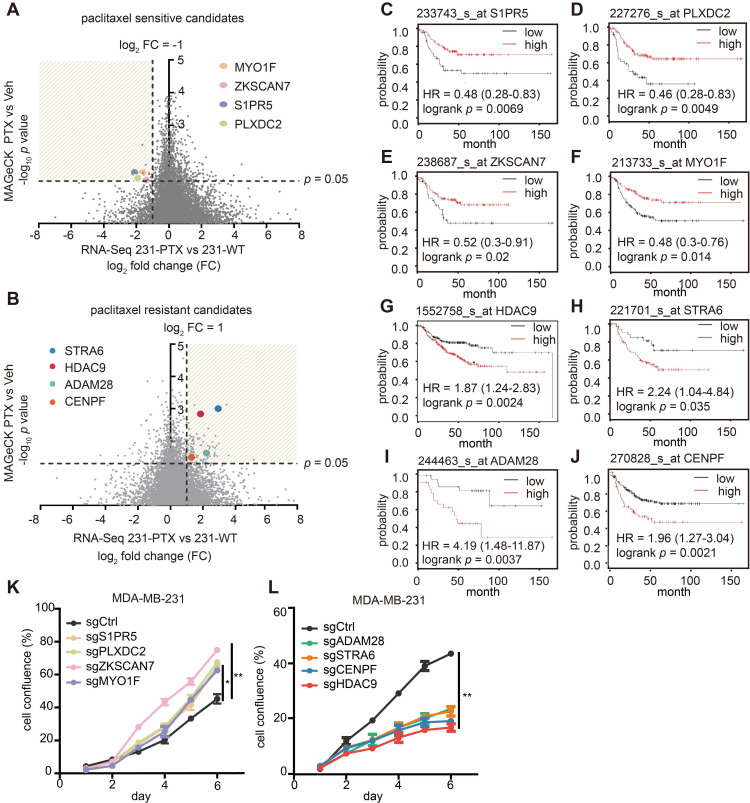
** Paclitaxel-sensitive/resistant candidates and their clinical prognostic values in breast cancer.** (A-B) Volcano plot displays gene distribution of paclitaxel-sensitive (A) paclitaxel-resistant (B) candidates. X-axis represents log_2_-fold change of 231-PTX versus 231-WT and Y-axis represents log_10_ *p* value of CRISPR/Cas9-positive (A) / (B) -negative screening. (C-F) Kaplan-Meier analysis of paclitaxel-sensitive candidates. Relapse-free survival Kaplan-Meier plots were based on gene expression, and the auto-select best cut-off was used to sort patients (*p* < 0.05). (G-J) Kaplan-Meier plot of paclitaxel-resistant candidates. Relapse-free survival Kaplan-Meier plots were based on gene expression, and the autos-elect best cut-off was used to sort patients (*p* < 0.05). (K-L) Cell growth after individual gene knock-out with single guide (sg) RNA was evaluated following treatment with 1 nM paclitaxel or DMSO for 6 d (*: *p* < 0.05; **: *p* < 0.01).

Transcriptome analysis revealed significant changes in gene expression within paclitaxel-resistant cell lines. In the meantime, the readout of a CRISPR/Cas9 knockout screen showed that paclitaxel-exposed cells demonstrated *de novo* gene changes. These two methods provided different perspectives for selecting paclitaxel-associated candidate genes, and both techniques were used for “paclitaxel-sensitive candidates,” which were genes with fold-change ≤ 0.5 in paclitaxel-tolerant cells, and sgRNA depletion with *p* < 0.05 in paclitaxel-treated cells, when compared with their respective controls (**Figure 2A**). Similarly, “paclitaxel-resistant candidates” were expected to meet the reverse requirements (**Figure 2B**).

Among hundreds of qualified genes, the association of clinical prognosis with gene expression was the last, but not the least, criteria. The KM plotter database, one of the largest breast cancer public databases with clinical prognoses, was used for this selection. Ultimately, four genes-S1PR5, PLXDC2, ZKSCAN7 and MYO1F were considered “paclitaxel-sensitive candidates” (**Figure 2C-F**), “paclitaxel-resistant candidates” were determined as four additional genes: HDAC9, STRA6, ADAM28 and CENPF (**Figure 2G-J**). The detailed RNA-Seq result and CRISPR analysis result of these genes were updated in **Table S4-5**. Real-time PCR was performed to validate differential gene expression in 231-PTX and 231-WT samples, where the results demonstrated a concordance between mRNA expressions of paclitaxel-sensitive or paclitaxel-resistant candidates with their RNA sequencing results (**Figure S2A-H**). Next, cell proliferation assay was carried out to evaluate how single-gene knockout influenced cell growth in PTX and Veh-treated cells. The result showed that single-gene knockout significantly increased (**Figure 2K**) or inhibited cell proliferation (**Figure 2L**) in the presence of paclitaxel, consistent with the screening results. Finally, after the combined analyses and verification, six candidates: S1PR5, MYO1F, PLXDC2, HDAC9, ADAM28 and STRA6, were identified as potential driver genes contributing to the paclitaxel effect. Thus, this integrated analysis effectively selects the paclitaxel-response candidates and represents a potential approach for exploring drug-specific driver genes.

### Truncated HDAC9 is the dominant isoform in paclitaxel-resistant cells

**Figure 3:**
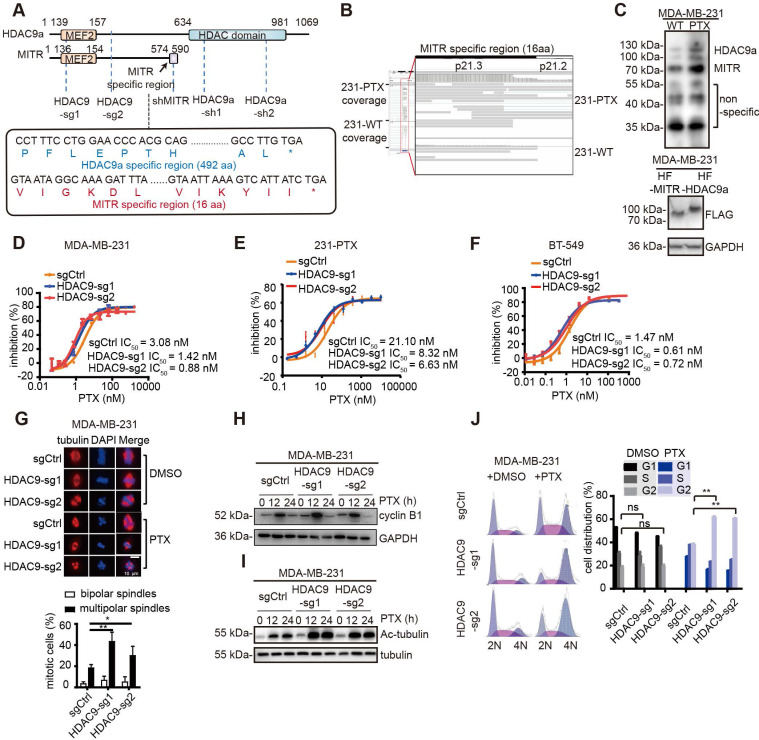
** HDAC9 plays a critical role in paclitaxel resistance.** (A) Schematic illustration of HDAC9 isoforms, as well as regions with sgRNA knockout and shRNA knockdown. The specific MITR region includes the C terminus of 16 amino acids, and the specific HDAC9a region starts from amino acid 609. (B) IGV screenshot of MITR and HDAC9a specific region in 231-PTX and 231-WT. (C) Western blotting of HDAC9 isoforms (MITR and HDAC9a) in 231-PTX and 231-WT cells (left), along with HDAC9 isoforms detected by FLAG-tag in MDA-MB-231 cells transfected with MITR and HDAC9a pdest-HA-FLAG vectors (right). (D-F) Cytotoxicity assay in HDAC9 knockout and control MDA-MB-231, 231-PTX and BT-549 cells treated with 3-serial-diluted paclitaxel dose for 72 h. The concentration of paclitaxel for half of the cell viability (IC_50_) was calculated (HDAC9-sg1 & HDAC9-sg2 vs sgCtrl: *p* < 0.05). (G) Representative confocal images of endogenous α-tubulin in HDAC9 knockout and control MDA-MB-231 cells at the middle of the mitotic period post-treatment with 1 nM paclitaxel (PTX) or DMSO for 48 h. Bar scale is 10 µM. Nucleus was stained with DAPI. Comparison of multipolar and bipolar spindle foci in HDAC9 sgRNA and control cells subject to paclitaxel treatment in three independent experiments (**: *p* < 0.01; *: *p* < 0.05). (H-I) Western blot images of cyclin B1 (H) and acetylated tubulin (I) in HDAC9 knockout and control MDA-MB-231 cells treated with 10 nM PTX or DMSO for different lengths of time. (J) Representative images of cell cycle in HDAC9 knockout and control MDA-MB-231 cells, treated with 10 nM PTX or DMSO for 12 h. Flow cytometry analysis of cell phase distribution was quantified, and each cell phase of different groups was measured and compared using two-sided Student's test (ns: *p* ≥ 0.05; **: *p* < 0.01). All data are expressed as mean ± sem.

The histone deacetylase (HDAC) family members, especially those in the HDAC Ia subgroup, have been reported to have great significance in drug resistance owing to their deacetylation activities. However, the function of HDAC9 in drug resistance has rarely been investigated. Moreover, HDAC9 has many variants, which can mainly be categorized into two groups: complete isoform HDAC9a, containing 24 exons, and the truncated isoform MITR with 13 exons (**Figure 3A**). The subcellular localization of ectopically expressed HDAC9a and MITR in MDA-MB-231 cells demonstrated that both FLAG-tagged HDAC9a and MITR were mainly within the nuclei (**Figure S3A**). Therefore, we focused on HDAC9, a member of the class Ⅱa HDACs, for further investigation. The IGV (Integrative Genomics Viewer) snapshots displayed that more assemblies covering the truncated HDAC9 (MITR) region in 231-PTX cells were present compared to 231-WT cells (**Figure 3B**). However, fewer assemblies were found for the HDAC9a region, compared to MITR, in both 231-PTX and 231-WT cells, with no significant differences between the groups (**Figure S3B**). Further analysis revealed HDAC9 isoforms being able to be detected through RNA sequencing, as well as the truncated isoforms being expressed at much higher levels, compared to the full-length isoforms, in both 231-PTX and 231-WT cells (**Figure S3C**). Next, we analyzed the protein expression of the two kinds of HDAC9 isoforms in 231-PTX and 231-WT cells using western blotting. As shown in **Figure 3C**, the truncated HDAC9 isoform MITR was significantly elevated in paclitaxel-resistant MDA-MB-231 cells, suggesting its enrichment in the paclitaxel-resistant cells. Since the isoform distribution was demonstrated in the bands, even non-specific ones, we further validated HDAC9 knockout MDA-MB-231 cells band distribution (**Figure S3D**). HDAC9a and MITR expression in TNBC cell lines was examined using specific antibodies, where the result showed that BT-549 cells had the highest expression level for both HDAC9a and MITR isoforms (**Figure S3E**). Thus, BT-549 cells were selected as the second cell line for experimentation.

### Suppression of HDAC9 augmented paclitaxel-mediated cytotoxic effects

To explore the role of HDAC9 in paclitaxel resistance, two sgRNAs were stably expressed in both parental and paclitaxel-resistant MDA-MB-231 and BT-549 cells, as well as in 231-PTX cells (**Figure S3F-H**). By targeting the common regions of HDAC9a and MITR, these two sgRNAs suppressed the expression of both full-length and truncated isoforms. Next, we conducted both *in vitro* and *in vivo* paclitaxel-mediated cytotoxicity assays. *In vitro* HDAC9 knockout resulted in a 3-4 fold lower IC_50_ in the paclitaxel-resistant group, vis-à-vis the control, in all cell lines (**Figure 3D-F**). Likewise, *in vivo* experiments in mice carrying MDA-MB-231 xenograft tumors with knocked-out HDAC9 exhibited significantly reduced tumor volumes post-paclitaxel treatment, compared to tumor-bearing mice with control sgRNA (**Figure S3I and S3J**).

After ensuring HDAC9 knockout phenotypes both *in vitro* and *in vivo*, we explored the impact of HDAC9 knockout on paclitaxel-mediated tubulin changes and cell cycle arrest. It has been reported that paclitaxel treatment stabilizes microtubules during mitotic division, causing bipolar spindle destruction and promoting abnormal multipolar spindle formation. Immunofluorescent staining of α-tubulin in paclitaxel-treated, HDAC9 knockout-MDA-MB-231 cells revealed remarkably more multipolar spindles versus control cells subjected to the same treatment (**Figure 3G**). Given the fact that paclitaxel inhibits bipolar spindle formation, the cells ended up being blocked in the G2 cell cycle phase, therefore eventually proceeding toward apoptosis. Western blotting of cyclin B1, a G2 phase marker, revealed higher levels in HDAC9 knock-out, paclitaxel-exposed cells, compared to non-knockout control cells (**Figure 3H**). Similar results were observed in BT-549 cells (**Figure S3K**).

Another method to assess microtubule stabilization is to test tubulin acetylation levels 32. After paclitaxel treatment, MDA-MB-231 and BT-549 cells with HDAC9 knock-out exhibited significantly increases in acetylated tubulin compared to non-knockout control cells (**Figure 3I and Figure S3L**). The cell cycle distribution in different MDA-MB-231 cell groups was evaluated with flow cytometry, following paclitaxel treatment. The results showed significant G2 phase blockage in HDAC9 knockout, paclitaxel-treated cells, while non-knock-out cells had normal cell cycle phase distribution (**Figure 3J**). These results suggested that HDAC9 knockout improved cell sensitivity to paclitaxel treatment by impairing tubulin stability and strengthening cell cycle arrest.

### MITR modulates paclitaxel resistance in TNBC cells

**Figure 4:**
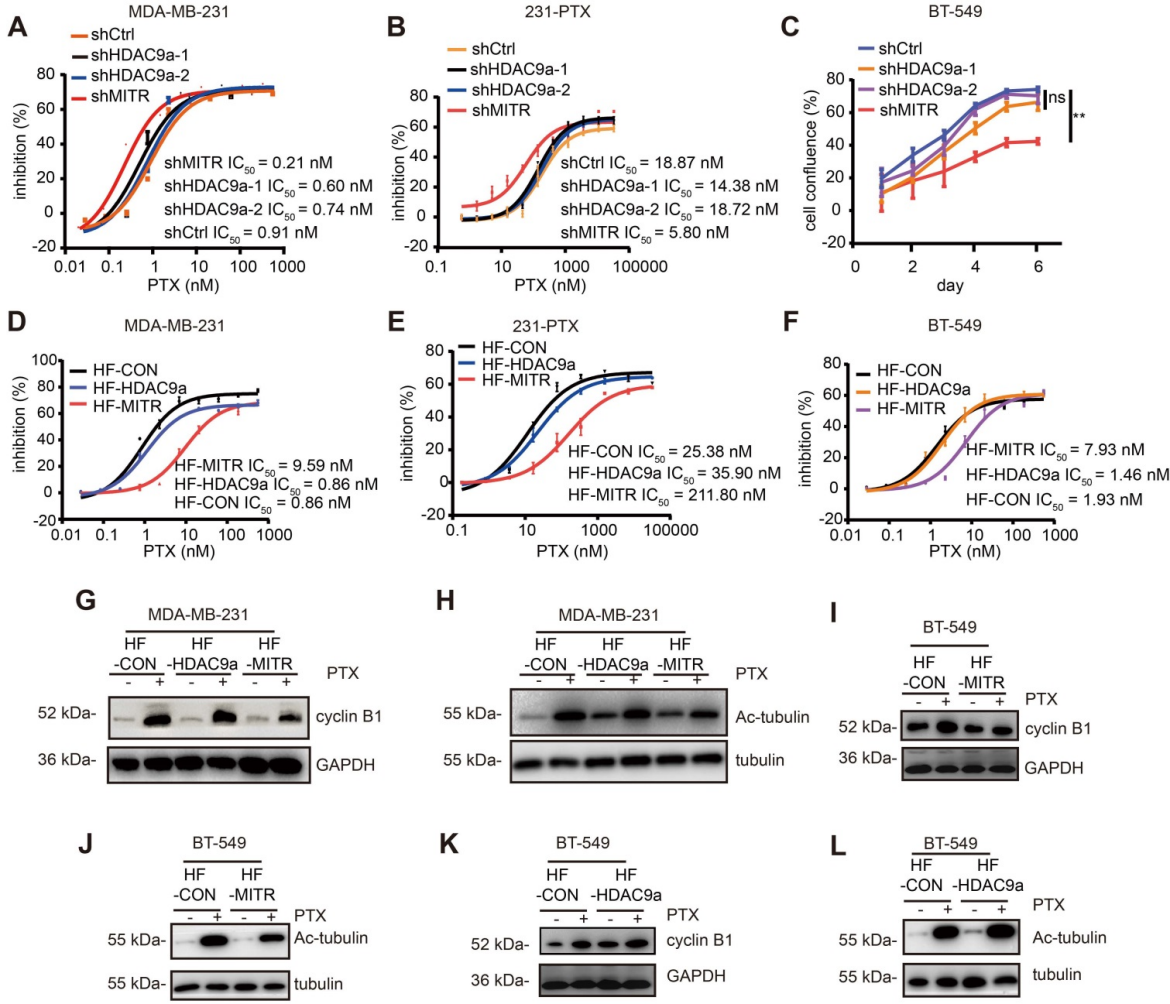
** Truncated HDAC9 isoform (MITR) induced paclitaxel resistance in breast cancer.** (A-B) Cell growth inhibition of MDA-MB-231 and 231-PTX cells transfected with shMITR or shHDAC9a, followed by treatment with different paclitaxel doses for 72 h. IC_50_ values were calculated and compared (shMITR vs shCtrl: *p* < 0.05, shHDAC9a-1 & shHDAC9a-2 vs shCtrl: *p* > 0.05). (C) BT-549 cells were transfected by shMITR or shHDAC9a, treated with 1 nM paclitaxel for 6 d, and cell growth inhibition was examined (shMITR vs shCtrl: p < 0.05, shHDAC9a-1 vs shCtrl: *p* > 0.05, shHDAC9a-2 vs shCtrl: *p* > 0.05). (D-F) MDA-MB-231, 231-PTX and BT-549 cells stably overexpressing MITR (HF-MITR), HDAC9a (HF-HDAC9a), and empty vector as control (HF-CON), and subject to gradient doses of paclitaxel for 3 d. IC_50_ was analyzed using Student's two-sided t-test (HF-MITR vs HF-CON: *p* < 0.05, HF-HDAC9a vs HF-CON: *p* > 0.05). (G-H) Western blot analysis in MITR or HDAC9a-overexpressed and control MDA-MB-231 cells under paclitaxel treatment, with cyclin B1 and acetylated tubulin antibodies. (I-L) Western blot analysis of cyclin B1 and acetylated tubulin in BT-549 overexpressed with MITR, HDAC9a and control cells treated with paclitaxel.

To elucidate which HDAC9 isoform plays a dominant role in paclitaxel resistance, we constructed shRNAs that stably knocked down each HDAC9 isoform type. The knockdown efficiency of shRNAs in MDA-MB-231, 231-PTX, and BT-549 cells was assessed by western blotting (**Figure S4A-C**). Subsequently, MDA-MB-231 and 231-PTX cells transfected with shMITR, shHDAC9a-1, shHDAC9a-2, and their respective controls were treated with gradient concentrations of paclitaxel. Interestingly, MITR knockdown led to a 3-fold lower IC_50_ value than that in control cells, whereas HDAC9a-1 or -2 knock-down had no significant effect on cell growth by paclitaxel (**Figure 4A**), vis-à-vis that in 231-PTX cells (**Figure 4B**). Growth assays were also performed in BT-549 cells transfected with shMITR, shHDAC9a-1, and shHDAC9a-2. Significantly slower cell growth was observed in shMITR-transfected cells, but not in shHDAC9a-1 or -2 transfected cells (**Figure 4C**). On the other hand, the MDA-MB-231, 231-PTX and BT-549 cells were transfected by exogenous lentiviruses, carrying MITR and HDAC9a sequences with an HA-FLAG (HF) tag at their N-terminus and the over-expression of HA-FLAG tagged MITR and HDAC9a were confirmed by western blotting (**Figure S4D-E**). Elevated MITR expression resulted in a 10-fold increase in IC_50_ value, while HDAC9a overexpression only slightly affected IC_50_ in paclitaxel-treated MDA-MB-231 cells (**Figure 4D**). Similar results were obtained in 231-PTX and BT-549 cells, which exhibited a 4-8 fold increase in IC_50_ compared HF-MITR cells to control cells, while no change in IC_50_ was detected with HDAC9a overexpression (**Figure 4E and 4F**). Western blotting revealed that MITR overexpression partially attenuated paclitaxel-induced up-regulation of cyclin B1 and acetylated tubulin, while HDAC9a overexpression showed minimal effects on cyclin B1 and acetylated tubulin protein expression (**Figure 4G and 4H**). Similar results were obtained in BT-549 cells, showing MITR overexpression partially decreased paclitaxel-induced up-regulation of cyclin B1 and acetylated tubulin, while HDAC9a overexpression did not change the expression of both proteins (**Figure 4I-L**). These results collectively demonstrated that MITR, the truncated isoform of HDAC9, played a critical role in modulating paclitaxel resistance.

### IL11 and its downstream JAK/STAT3 signaling are involved in MITR-mediated paclitaxel resistance

**Figure 5:**
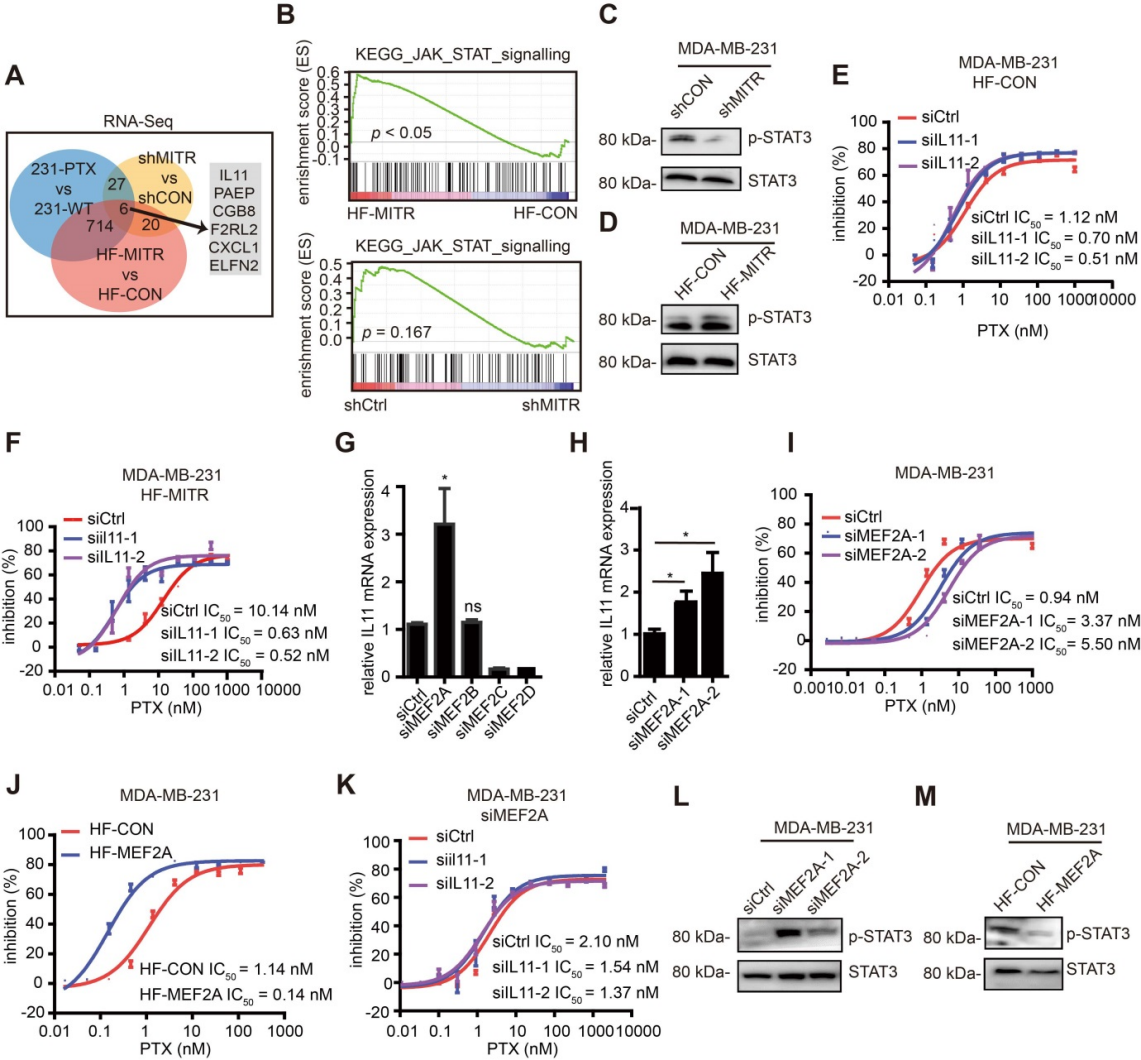
** MITR regulated IL11 and JAK/STAT3 signaling.** (A) RNA sequencing comparing 231-PTX to 231-WT, MDA-MB-231 with MITR knockdown (shMITR) to control (shCtrl), and MDA-MB-231 with overexpressed MITR (HF-MITR) to control (HF-CON). Venn diagram displayed the number of significant genes found between different combinations of two RNA-seq analyses, as well as those found between all 3 RNA-seq sets. Significant genes were selected based on log _2_-fold change ≥ 1 or ≤ -1. (B) GSEA enrichment analyses of KEGG JAK/STAT signaling pathway. Upper image shows HF-MITR versus HF-CON group, and lower image shows shMITR versus shCtrl group. (C-D) Phosphorylated STAT3 was blotted in shMITR (C), HF-MITR (D), and control MDA-MB-231 cells. (E-F) Cell growth inhibition curves of HF-CON and HF-MITR MDA-MB-231 cells with IL11 silencing in response to paclitaxel. IC_50_ values were analyzed (siIL11-1 & siIL11-2 vs siCtrl E: *p* > 0.05; F: *p* < 0.05). (G) Relative IL11 mRNA expression after silencing each of the MEF2 members in MDA-MB-231 cells (ns: *p* ≥ 0.05, *: *p* < 0.05). (H) After silencing MEF2A, relative IL11 mRNA expression was detected by RT-PCR in MDA-MB-231 cells (*: *p* < 0.05). (I-J) Cell growth inhibition to paclitaxel treatment was evaluated in MDA-MB-231 cells transduced by MEF2A siRNA (siMEF2A) and MEF2A vector (HF-MEF2A), compared to their respective controls. IC_50_ values were analyzed and compared (siMEF2A-1 & siMEF2A-2 vs siCtrl: *p* < 0.05; HF-MEF2A vs HF-CON: *p* < 0.05). (K) si-MEF2A MDA-MB-231 with IL11-silencing or control cells were evaluated for cell growth inhibition, following treatment with different paclitaxel doses (siIL11-1 & siIL11-2 vs siCtrl: *p* > 0.05). (L-M) Western blot analyses of phosphorylated STAT3 in MDA-MB-231 cells with MEF2A silencing (L) or MEF2A overexpression (M).

To understand the molecular mechanisms involved in MITR-mediated paclitaxel resistance, we analyzed the transcriptomes of 231-PTX and 231-WT cells with stable MITR knockdown or overexpression, along with their corresponding controls (**Figure 5A**). Interestingly, the JAK/STAT3 signaling pathway was significantly enriched in the MITR-overexpressing transcriptome (**Figure 5B**). Although GSEA analysis for shMITR group is not as significant as that for the HF-MITR over-expressing group, JAK-STAT signaling still ranked among the top 20 KEGG pathways (**Figure S5A**). We hypothesized that MITR may up-regulate Interleukin11 (IL11), thus regulating the JAK/STAT3 pathway.

ILs have been shown to play a role in cancer drug resistance. One IL, IL6 is an oncogenic target that dominates the downstream JAK/STAT3 pathway 33. Several researchers have found that IL11, another member of the IL6 family, can also activate JAK/STAT3 pathway in some cancer types 34-36. Phosphorylated STAT3 levels and IL11 expression were elevated or decreased, in line with MITR levels (**Figure 5C-D and S5B-D**). In order to confirm IL11 was the downstream mediator of MITR, we silenced IL11 in MITR-overexpressing MDA-MB-231 and the corresponding control cells, followed by paclitaxel treatment (**Figure S5E**). As shown in **Figure 5E**, silencing IL11 in control cells slightly decreased IC_50_. However, the higher IC_50_ in MITR-overexpressing MDA-MB-231 was completely suppressed by IL11 silencing (**Figure 5F**). Similarly, when investigating the role of IL11 in MITR overexpressing 231-PTX cells, we found that silencing IL11 inhibited cell proliferation in paclitaxel-treated cells, compared to controls (**Figure S5F**). All these results implied that MITR induced paclitaxel resistance in an IL11 dependent manner.

### MITR repressively interacts with MEF2A to transcriptionally up-regulate IL11 expression

Since MITR is the truncated, enzymatically inactive isoform of HDAC9, we explored whether it directly or indirectly regulated IL11 expression at the transcriptional level. MITR was firstly reported as the interacting transcription factor of MEF2, and its direct binding to its N-terminal domain resulted in MEF2 transcriptional repression 11. It is therefore plausible that MEF2 family members may play a role in the MITR/IL11 axis. We used the PROMO transcriptional prediction website, which identified MEF2A as IL11-related transcription factor, leading us to hypothesize that MEF2, when repressed by MITR through its binding to the N-terminal protein domain, inhibited IL11 downstream expression. To confirm this hypothesis, four MEF2 family members were individually silenced in MDA-MB-231 cells (**Figure S5G**), and the IL11 mRNA expression was detected by qPCR. The results demonstrated that only MEF2A had negative correlations with IL11 expression (**Figure 5G**), where MEF2A silencing (**Figure S5H**) significantly up-regulated IL11 (**Figure 5H**). Furthermore, this silencing resulted in 3-5 fold increase for IC_50_ in paclitaxel-treated MDA-MB-231 cells (**Figure 5I**). On the other hand, MEF2A over-expression in MDA-MB-231 cells (**Figure S5I**) was associated with dramatically decreased paclitaxel IC_50_ values (**Figure 5J**). We also performed functional rescue assays to silence IL11 expression in MEF2A-siliencing MDA-MB-231 cells. As expected, the increased paclitaxel IC_50_ by MEF2A-siliencing was restored by IL11 inhibition (**Figure 5K**). Moreover, phosphorylated STAT3 was up-regulated when MEF2A was knocked down, and down-regulated when MEF2A was over-expressed (**Figure 5L and 5M**).

**Figure 6:**
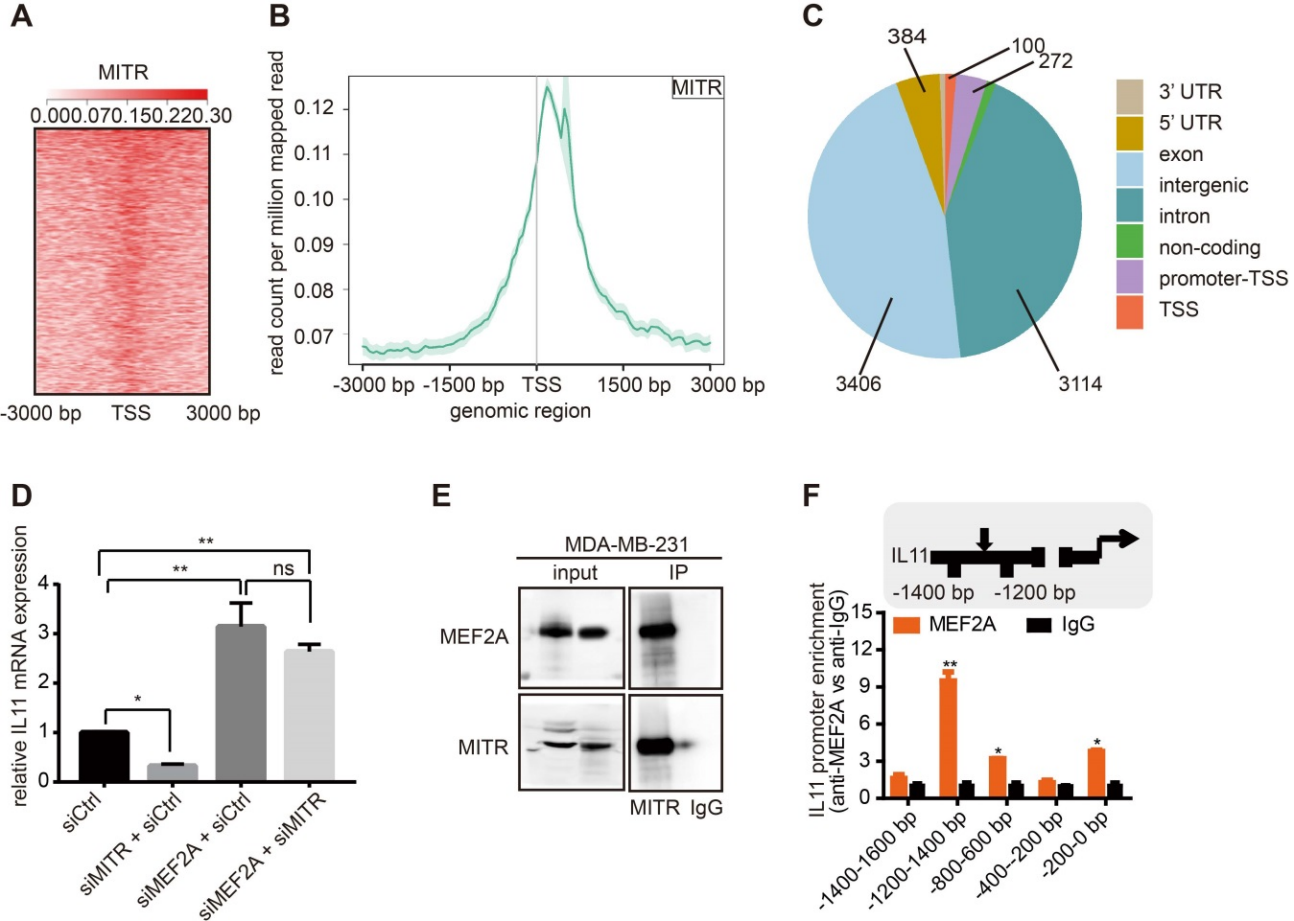
** MITR ChIP analysis of MITR, MEF2A and IL11 interactions.** (A) Heat-map showing peak MITR distribution being localized around the TSS region. (B) Cumulative curve of peak MITR distribution, with the highest point near the TSS region. (C) Pie chart exhibiting the percentages of 7 peaks. (D) Relative IL11 mRNA expression in MDA-MB-231 cells silenced by siMEF2A, siMITR, siMITR + siMEF2A, and their respective controls (ns: *p* ≥ 0.05, **: *p* < 0.01, ***: *p* < 0.001). (E) MEF2A-MITR complexes were immunoprecipitated using 1 µg of anti-MITR or anti-IgG control antibody. The same amount of cell lysates in the input group was blotted by anti-MITR and anti-MEF2A antibodies. (F) Amplification of the IL11 promoter region by real-time PCR. Chromatin was immunoprecipitated (ChIP) from MDA-MB-231 cells transfected with MEF2A vector, using an anti-FLAG antibody. Normal rabbit IgG was used as a negative control, while U6 primers were used as a negative control. The MEF2 binding site, the amplified region and the TSS are indicated in the ideogram. *: *p* < 0.05, **: *p* < 0.01 vs all the other regions.

MITR may regulate IL11 via MEF2A, or by directly binding to the IL11 promoter region. To distinguish between these possibilities, we performed MITR ChIP sequencing in MDA-MB-231 cells using the anti-MITR antibody. Over 7,000 peaks were detected in MITR ChIP analyses. We found that MITR occupied region was enriched around the TSS region (**Figure 6A and 6B**). Giorgio *et al* had analyzed HDAC9 ChIP sequencing and found that distal intergenic regions occupied more than 70% of all the peaks 37. We also found that the main components of peaks in MITR ChIP-seq were in intergenic and intron regions (**Figure 6C**), suggesting that MITR might also occupy the distal regulative elements. As there was no IL11 peak in the MITR ChIP-seq, we have less evidence to conclude that MITR directly bound to IL11. To verify whether MITR interacts with MEF2A to regulate IL11 expression, we designed the rescue experiments. As shown in **Figure 6D**, knockdown of MEF2A caused IL11 up-regulation, while silencing MITR suppressed IL11 expression in MDA-MB-231 cells. However, IL11 suppression, triggered by MITR silencing, was abolished when MEF2A was simultaneously silenced. We also performed the Co-IP assay between MITR and MEF2A, and the results suggested direct protein-protein interactions between MITR and MEF2A (**Figure 6E**). In addition, the MEF2A ChIP assay was performed by FLAG-tag using MEF2A-overexpressing MDA-MB-231 cells. We detected MEF2A enrichment at the IL11 promoter region, implying that it might transcriptionally regulate IL11 (**Figure 6F**). All evidence thus suggested that MITR might interact with MEF2A at the transcriptional level to regulate IL11 expression.

### Clinical relevance of MITR /MEF2A/IL11 axis in TNBCs

**Figure 7:**
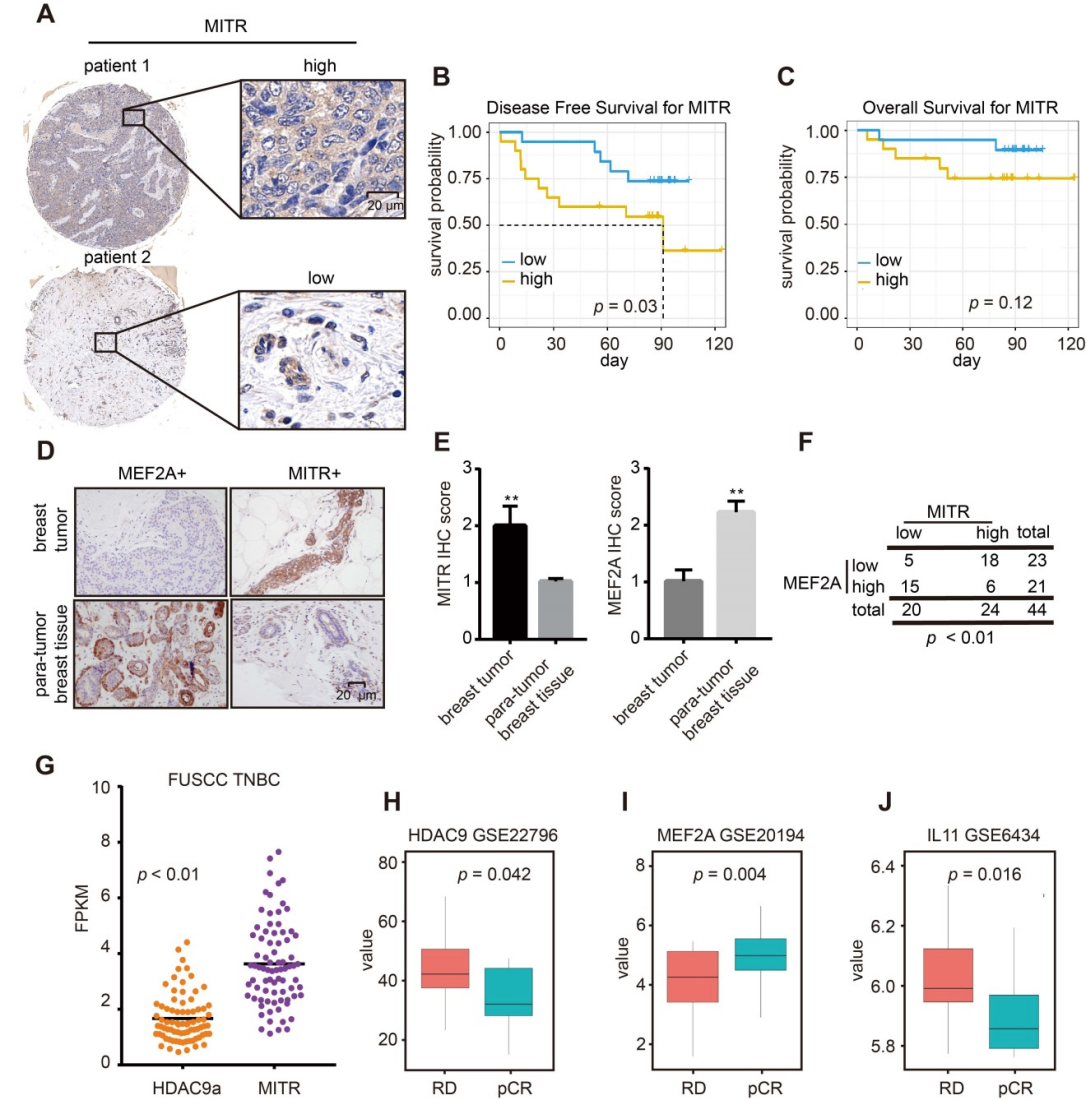
** IHC and clinical analyses of MITR and MEF2A.** (A) Representative images of immunohistochemically (IHC) staining for high and low MITR levels in triple negative breast cancer (TNBC) tissues. (B-C) Kaplan-Meier curves of overall survival and disease-free survival, related to MITR expression. (D) Representative IHC images of MEF2A and MITR in TNBC breast carcinoma and para-normal breast tissue. (E) Score quantification comparing MITR and MEF2A IHC scores between 44 pairs of TNBC tumor and para-tumor tissues (**: *p* < 0.01). (F) Patients were assigned into 4 groups, categorized by MITR and MEF2A IHC expression and analyzed by person Pearson's X² test. (G) The mRNA expression of MITR and HDAC9a in FUSCC TNBC database: two-tailed paired t-test was performed and the average fold-change, with respect to MITR and HDAC9a expression, was indicated. (H-J) mRNA expression levels of MITR (HDAC9), MEF2A and IL11 in TNBC patients with complete pathological cure (pCR) or residual disease (RD) after neoadjuvant chemotherapy from GEO databases.

To explore the clinical significance of the MITR (HDAC9)/MEF2A/IL11 axis in TNBCs, we performed immunohistochemical (IHC) analyses on 44 primary breast tumors and 4 noncancerous mammary controls with the MITR antibody. We quantified MITR expression in IHC images and classified samples into low and high MITR expression groups (**Figure 7A**). Survival analysis showed better disease-free survival with low MITR expression (**Figure 7B**), and similar trends were observed in the overall survival analysis (**Figure 7C**). Additionally, MITR was highly expressed in tumors but lowly expressed in non-tumorous tissues, while the opposite was found for MEF2A (**Figure 7D and 7E**). A significant negative correlation was found between MITR and MEF2A levels (*p* < 0.01, **Figure 7F**). We further analyzed HDAC9 mRNA transcripts in the TNBC samples from FUSCC TNBC dataset 38 and found MITR expression to be much higher than full-length HDAC9a isoform in tumors, consistent with mammary cancer cell line results (**Figure 7G**).

Since the majority HDAC9 was verified to be the MITR isoform, the MITR/MEF2A/IL11 axis may be prevalent in TNBCs. To validate the importance of this MITR-modulated axis in clinical tumors, we searched for GEO mRNA expression datasets with information on TNBC patients, treated with or without neoadjuvant chemotherapy containing paclitaxel. We evaluated patient prognosis and found significantly high MITR (HDAC9) and IL11 expression in pathologically-progressive cases, as well as remarkably low MEF2A expression in patient tissues undergoing complete response (pCR) (**Figure 7H-7J**). Subsequently, we examined MITR and MEF2A associations with their downstream gene, IL11. We observed positive correlations between MITR (HDAC9) and IL11 expression, and negative correlation between IL11 and MEF2A/MITR (HDAC9) in TNBCs (**Figure S6A-C**). In addition, we used KM plotter public datasets to explore gene expression and patient prognosis. High MITR (HDAC9) and IL11 expression correlated with poor survival (**Figure 2G and S6D**), while the higher MEF2A expression was associated with favorable clinical outcomes for TNBC patients (**Figure S6E**). Taken together, MITR and IL11 may serve as negative prognostic markers in TNBCs, where higher levels may indicate worse responses to paclitaxel-containing chemotherapy. In contrast, MEF2A represents a positive prognostic value, and TNBC patients with higher MEF2A expression might benefit from paclitaxel treatment.

### Paclitaxel combined with ruxolitinib exhibits promising effects on MITR-enriched TNBC tumors

**Figure 8:**
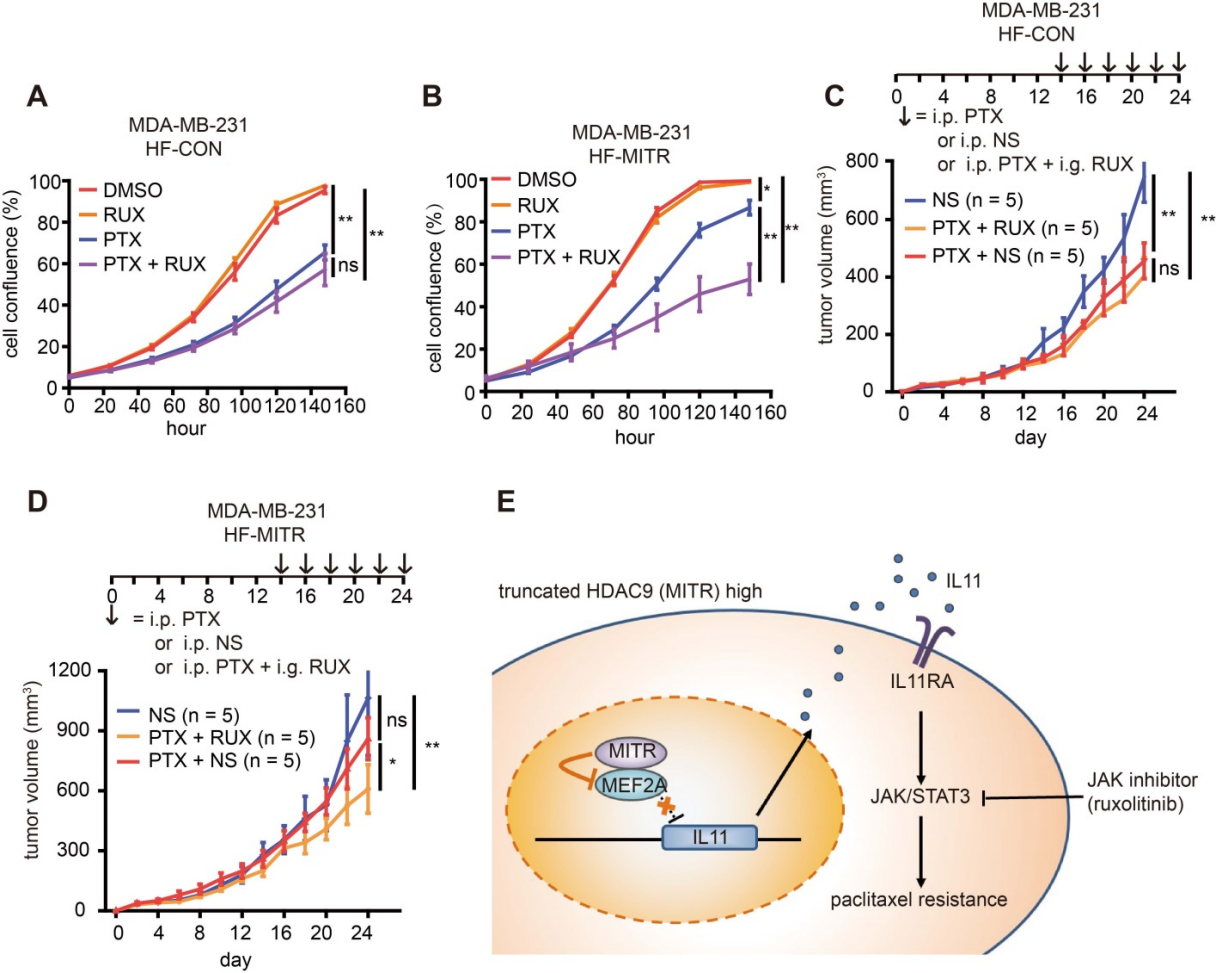
** Combination of paclitaxel and ruxolitinib treatment is efficient for tumor regression in TNBC with high MITR expression.** (A-B) Percentage of cell confluence in HF-CON (control) and HF-MITR (MITR-overexpressing) MDA-MB-231 cells treated with 1 nM paclitaxel (PTX), 10 nM ruxolitinib (Rux), 1 nM paclitaxel plus 10 nM ruxolitinib (PTX + Rux), or DMSO for 160 h. Different groups were compared with t-test (ns: *p* ≥ 0.05, *: *p* < 0.05, **: *p* < 0.01). (C-D) Volume of HF-CON and HF-MITR MDA-MB-231 tumors after treatment with 5 ng/kg paclitaxel (PTX + NS), 100 ng/kg ruxolitinib + 5 ng/kg paclitaxel (PTX + RUX), or the same volume of normal saline (NS, ns: *p* > 0.05, *: *p* < 0.05, **: *p* < 0.01). Each group contains 5 BALB/c nude mouse. (E) Proposed model of MITR-induced paclitaxel resistance in TNBCs. MITR upregulates IL11 expression and subsequently activates JAK/STAT3 signaling, thus inducing paclitaxel resistance. The repressive interaction of MITR with MEF2A to eliminate MEF2A transcriptional suppression on IL11 is believed to be the mechanism of MITR-induced paclitaxel resistance. The combination of JAK inhibitor ruxolitinib with paclitaxel restores paclitaxel efficacy for the treatment of TNBC patients with higher MITR expression.

The ultimate aim of exploring MITR-mediated paclitaxel resistance is to identify potential paclitaxel tolerance in TNBC patients before treatment, and possible alternatives for targeted personalized therapy. Since MITR lacks the common target site for HDAC inhibitors, such as TSA or TMP195, we used the pathway inhibitor ruxolitinib, which mainly inhibits JAK1 and JAK2. We observed a strong effect of the combined treatment with ruoxlitinib and paclitaxel in MITR-overexpressing MDA-MB-231 and BT-549 cells. In MDA-MB-231 cells, we performed cell viability assays in the presence of 1 nM paclitaxel with 10 nM ruoxilitinib and did not observe additional cytotoxic effects of ruoxilitinib on cell growth (**Figure 8A**). However, ruoxlitinib could reverse MITR-induced paclitaxel resistance in MITR over-expressing cells (**Figure 8B**). In BT-549 and 231-PTX cells, paclitaxel and ruoxolitinib combined treatment reduced IC_50_ values in the MITR over-expressing group to approximately the same levels as in the control group (**Figure S7A-C**). These results suggested that JAK1/2 inhibition by ruxolitinib sensitized cancer cells to paclitaxel treatment by antagonizing the effect of MITR. *In vivo*, we found that paclitaxel mono-therapy (PTX + saline (NS)) dramatically suppressed tumor growth in MDA-MB-231 xenograft mouse model compared to NS controls. However, combined paclitaxel and ruoxolitinib treatment (PTX + RUX) did not obtain additional benefits (**Figure 8C**)**.** In MITR-overexpressing groups, tumors were not significantly suppressed under paclitaxel mono-therapy (PTX + NS) compared to NS group, while the effect of paclitaxel was dramatically improved when combined with ruoxolitinib (PTX + RUX, **Figure 8D**). These results suggested that JAK1/2 inhibition by ruxolitinib sensitized cancer cells to paclitaxel treatment by antagonizing the effect of MITR.

Thus, our study uncovered a MITR/MEF2A/IL11 axis, which modulates paclitaxel resistance by activating JAK/STAT3 signaling in TNBCs. As illustrated in **Figure 8E**, higher MITR expression counteracts MEF2A to increase IL11 transcription. The increased expression and secretion of IL11 activates the downstream JAK/STAT3 pathway, ultimately leading to elevated paclitaxel resistance. Thus, JAK1/2 inhibition would be a potentially attractive target to reverse the MITR-induced paclitaxel resistance in TNBCs.

## Discussion

Since the first-line chemotherapy regimen for TNBC patients includes paclitaxel, it is necessary to explore the driver genes responsible for poor paclitaxel response in TNBC patients. High-throughput screening techniques are considered helpful in identifying novel mechanisms of drug resistance 39. In this study, we conducted genome-wide CRISPR screening following paclitaxel treatment, and integrated the transcription profile of paclitaxel-resistant cells to identify novel candidates. HDAC9 was detected as a promising target in regulating paclitaxel resistance. Suppression of HDAC9 expression led to microtubule dysfunction and G2 cell phase blockage, thus increasing cell apoptosis in paclitaxel-treated TNBC cell lines. Notably, MITR, the non-deacetylating truncated HDAC9 isoform, was responsible for TNBC paclitaxel resistance, via the expression of that isoform being tightly associated with the downstream IL11/JAK/STAT3 signaling pathway. Mechanistically, we found MEF2A directly bound to IL11 promoter to suppress IL11 expression. High MITR expression abolished the repressing effect of MEF2A, ultimately leading to the IL11 up-regulation and JAK/STAT3 signaling pathway activation. We also found that a JAK inhibitor could restore the paclitaxel response abolished by MITR both *in vitro* and *in vivo*. Thus, MITR could be a promising predictive biomarker for paclitaxel response, and a therapeutic target for TNBC patients receiving combined treatment with paclitaxel and a JAK inhibitor.

HDAC9, which influences tubulin stability and cell cycle arrest, is reported to have two major isoforms. One isoform is the full-length form of HDAC9, with low expression in breast cancer cell lines. Despite having a binding domain for MEF2, HDAC9a has no effect on paclitaxel resistance, probably due to its deacetylation domain nullifying against any possible effects from the HDAC9a-MEF2 interaction. However, this hypothesis needs further validation. Several studies have reported the significance of cancer-implicated isoforms. Therefore, we analyzed HDAC9 isoforms and found that the truncated isoform MITR was involved in TNBC paclitaxel resistance. MITR lacks the C-terminal deacetylation domain and shows much higher expression in breast cancer cells than the full-length form. It was also reported to repress MEF2 function by directly binding to its N-terminus 40. MEF2 genes, in turn, encode transcription factors with both pro-oncogenic and tumor suppressive activities 41. MEF2A overexpression augmented paclitaxel response in TNBCs, suggesting that it acts as a suppressor for TNBC paclitaxel resistance.

Previous studies have reported on transcriptional regulation by HDAC-MEF2 complexes. Kong *et al* proposed that the intron domain of IL6 could recruit the HDAC-MEF2 repressive complex, which suppressed the pro-inframammary target gene, thereby enhancing chronic infections and cancer 42. Giorgio *et al* demonstrated that regions recognized by MEF2D/HDAC4/HDAC9 repressive complexes demonstrated associations as active enhancers for tumor growth, and many of them were bonded both by MEF2s and HDACs 37. In our study, we confirmed that MEF2A could directly bind to and suppress IL11. However, the detailed analysis of MEF2A and IL11 interactions needs to be further validated.

Another critical issue in the article is whether other HDAC family members influence the MITR-MEF2 complex, particularly with respect to IL11 upregulation. Here, we speculate that HDAC9 alone is responsible for IL11 increases in paclitaxel-resistant cells. Figure 3B displayed that the assemblies covering the truncated HDAC9 (MITR) region were far more than those in the full-length HDAC9 -specific region (HDAC9a), suggesting that MITR was the dominant isoform of HDAC9. Furthermore, RNA sequencing indicated that HDAC9 was enriched significantly in the 231-PTX cells, while other HDAC family members did not exhibit any differences in expression levels between 231-PTX and 231-WT (**Table S6**). These evidences supported that truncated HDAC9 (MITR) might be one of the strongest repressors against MEF2A to up-regulate IL11 expression in TNBCs. Moreover, the rescue experiment showed that MITR failed to induce IL11 when MEF2A was silenced simultaneously, indicating MITR might regulate IL11 expression at a MEF2A-dependent manner. Nevertheless, Verdin *et al* proposed that there are two possible mechanisms for class IIa HDACs in mediating downstream promoters. One mechanism is direct interaction, while the other is indirect recruiting co-regulators and interacted through MEF2s 43. It remains possible, and even likely, that MITR could release stronger repressors from MEF2A to regulate IL11 expression. Therefore, more investigations should be performed to clarify the underlying mechanism of MITR on IL11.

Paclitaxel is a tubulin-targeting drug, which interferes with microtubule dynamics and blocks mitosis. Previous studies of paclitaxel resistance typically focused on mechanisms such as tubulin mutation, MDR overexpression, and chromosomal instability. In the current study, we aimed to understand the mechanisms responsible for paclitaxel resistance involving JAK/STAT3 signaling. Previous studies have reported that STAT3 activation occurs through the IL6 cytokine family 44. Hartman and colleagues described that IL6 family members IL6 and IL8 are critical for resistance to paclitaxel, due to their stimulation of multiple pathways, including the JAK/STAT3 pathway 45. However, Bockhorn and colleagues illustrated the significance of another IL6 family member, IL11, in inducing paclitaxel resistance via activating the same aforementioned downstream pathway 46. JAK 1/2-mediated STAT3 phosphorylation leads to stable homodimer and heterodimer formation, which bind to specific promoters, resulting in aberrant gene expression with respect to regulating cell proliferation, differentiation, and apoptosis (*e.g.* Bcl2 and NF-κB) 47, 48. In our study, we found that HDAC9 expression variation was proportional to IL11 mRNA expression and STAT3 protein phosphorylation state. Thus, IL11, in contrast to IL6 and IL8, acts as a cytokine that activates the JAK/STAT3 pathway and mediates TNBC chemotherapy response. As an extracellular component, it is influenced by the tumor microenvironment, which may also contribute to paclitaxel resistance.

The pan-HDAC inhibitors, such as SAHA, and class IIa HDAC inhibitors, such as TMP195, target the deacetylation domain of HDACs. We chose the JAK1/2 inhibitor ruxolitinib for the combinational treatment used in this study. Ruxolitinib was approved by the FDA for patients with intermediate or high-risk myelofibrosis, based on the results of several randomized phase III trials 49. However, the phase Ⅱ trial of ruxolitinib in malignant TNBC patients failed 50. This could be due to the heterogeneity of TNBC consisting of several subpopulations. In our study, we found that MITR was the main isoform of HDAC9 in cultured mammary cancer cells and TNBC tumors. We verified that TNBC subpopulations with high MITR and IL11 expression likely develop aggressive tumors since their poor paclitaxel response. We predicted that in these high MITR/IL11 expression subpopulations, the combined treatment of ruxolitinib and paclitaxel, instead of paclitaxel monotherapy, would improve therapeutic efficacy.

In summary, our study emphasized the importance of MITR, the truncated deacetylation-lacking HDAC9 isoform. Our results revealed that the MITR/IL11 axis activates JAK/STAT3 signaling, thus inducing paclitaxel resistance in TNBCs. More importantly, we also provided evidence that a strategy of combining with JAK1/2 inhibitor ruxolitinib sensitizes cancer cells to paclitaxel treatment. The mechanistic insight into the MITR/MEF2A/IL11 axis provided by our *in vitro* and *in vivo* studies, together with the GEO mRNA expression datasets of TNBC patients is also of prognostic significance. Thus, our study has outlined a novel strategy for understanding chemotherapy resistance. In the future, it would be worthwhile to conduct a clinical trial with combined ruxolitinib and paclitaxel treatment for eligible TNBC patients.

## Supplementary Material

Supplementary figures.

Supplementary table S1, S2, S3, S4, S5, S6.
