# Supplementary material for: Truncated HDAC9 identified by integrated genome-wide screen as the key modulator for paclitaxel resistance in triple-negative breast cancer
Source: Theranostics. 2020 Sep 2;10(24):11092–109. doi: 10.7150/thno.44997 (PMC7532680; doi:10.7150/thno.44997)
Supplement: Supplementary file 1 — Supplementary figures. [file thnov10p11092s1.pdf]

Supplementary files

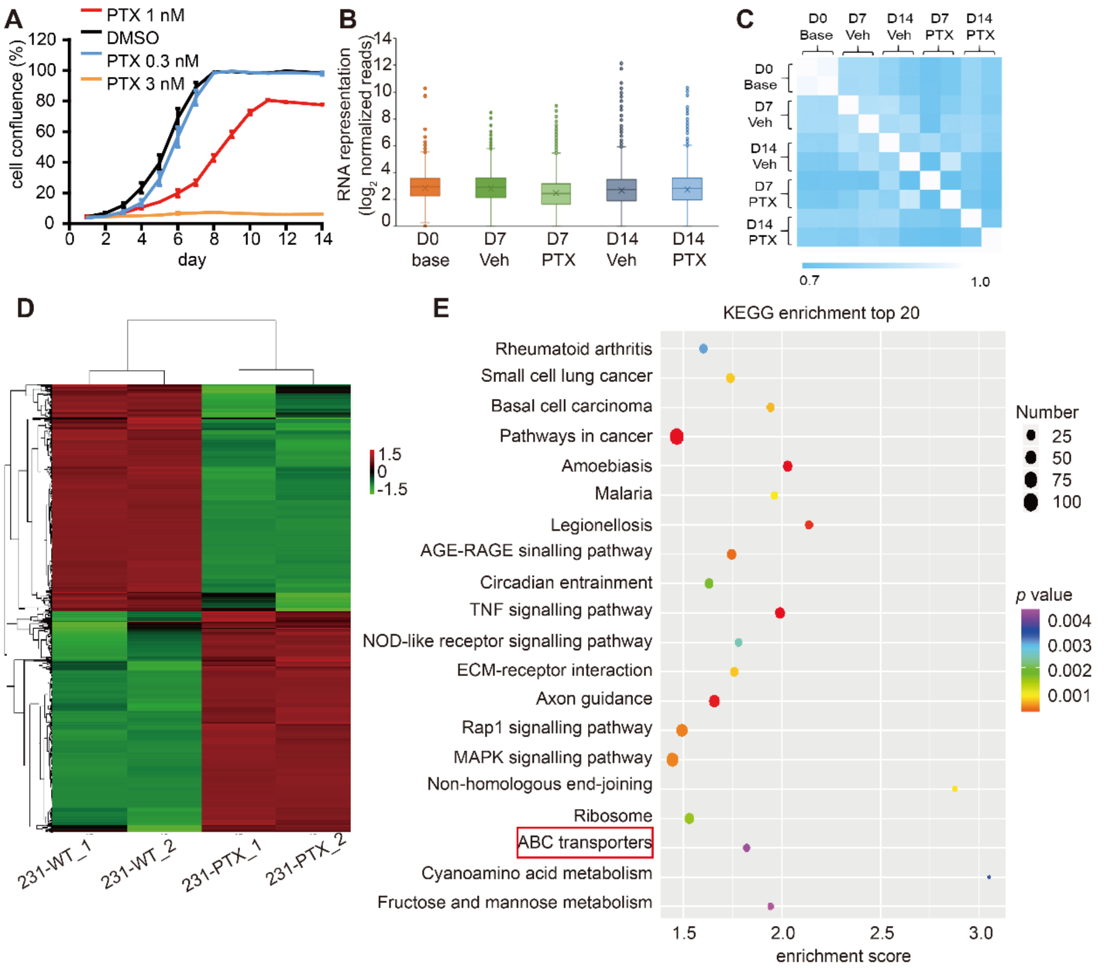

Figure S1. Gene heatmap and KEGG pathway clustering of RNA sequencing. (A) MDA-MB-231 growth curve with different doses of PTX and DMSO after 14 days of treatment. (B) sgRNA reads distribution of various samples: greater enrichment of sgRNA reads in D14 samples, with or without PTX treatment. (C) Correlation of sgRNA reads among different samples and replicates. High correlation of sgRNA reads between replicates, but low correlation of sgRNA reads between samples with or without PTX treatment at the same time point. (D) Heat map of gene profiles of two replicates of 231-PTX and 231-WT. (E) List of the top 20 KEGG pathways. The size of the bubble of each pathway represents the number of significantly expressed genes and the color of the bubble indicates the *p* value.

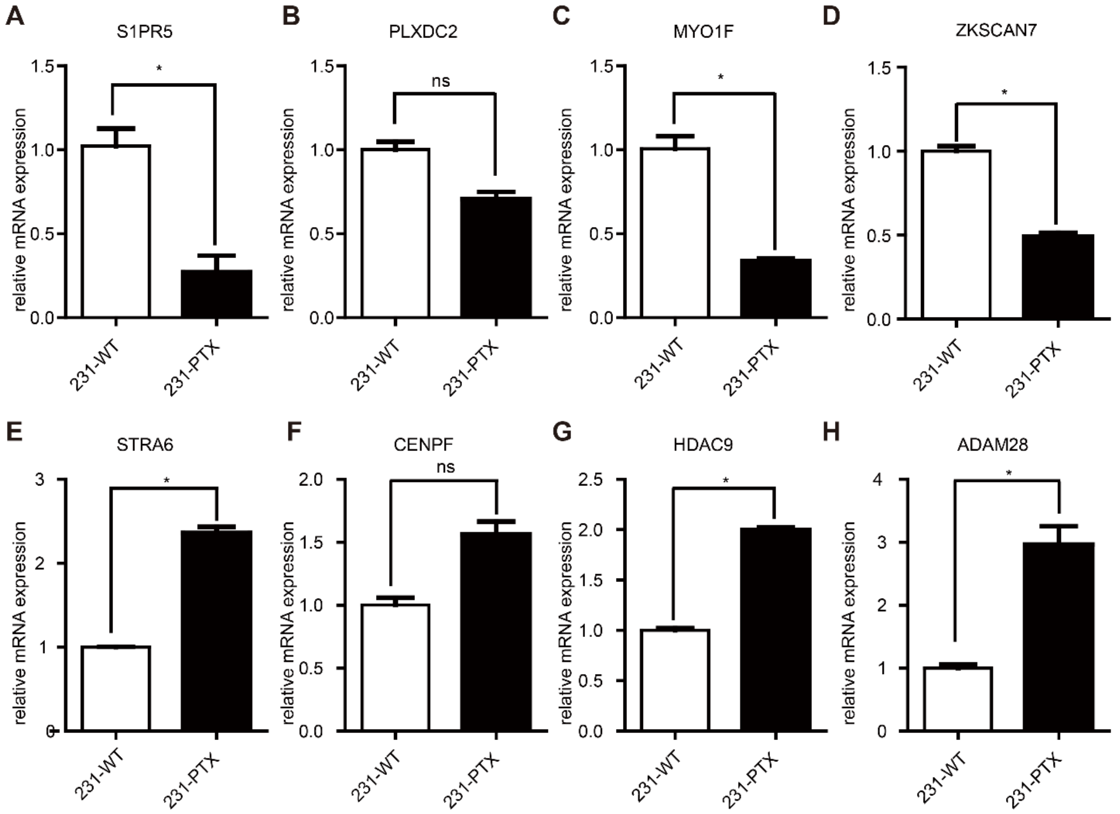

Figure S2. Validation of selected genes by RT-qPCR. (A-H) Histograms of mRNA relative expression comparing 231-PTX to 231-WT by RT-qPCR (*: $p < 0.05$; ns: $p \geq 0.05$).

-

-

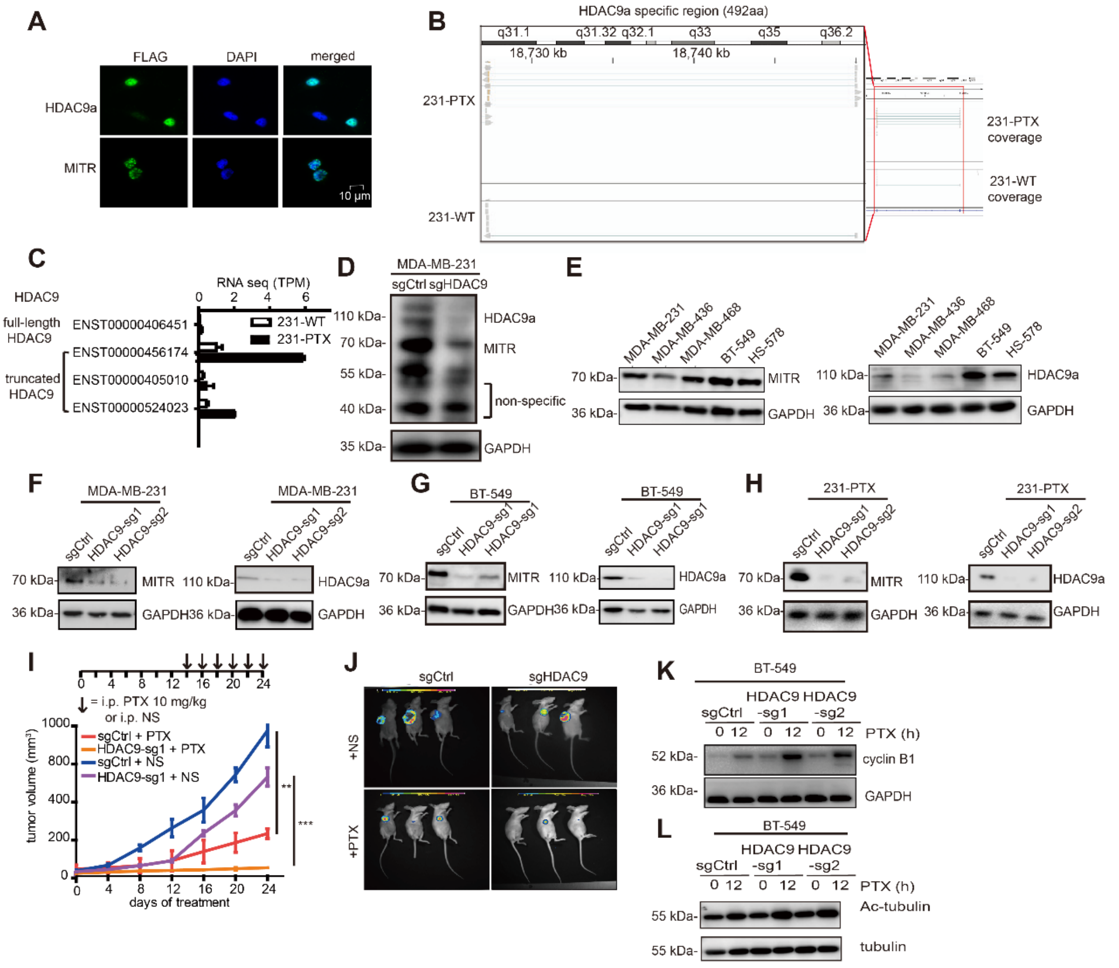

Figure S3. HDAC9 knockout BT-549 cells show a similar phenotype with MDA-MB-231 cells. (A)Immunofluorescence images of MDA-MB-231 transfected by HDAC9a and MITR plasmids. MDA-MB-231 cells were stably expressed flag-HDAC9a and flag-MITR as indicated and were stained with DAPI (blue) and FLAG antibody (green). (B) IGV view of HDAC9a coverage in MDA-MB-231 paclitaxel resistant cells (231-PTX) and parental cells (231-WT). (C) Expression of different HDAC9 transcripts in 231-PTX and 231-WT (*: $p < 0.05$). (D) Same amount of MDA-MB-231control and HDAC9 knockdown cell lysates were blotted by HDAC9 antibody (Thermo fisher PA5-11246), which displayed the position of HDAC9a, MITR and non-specific band. (E) Western blot of HDAC9 isoforms HDAC9a and MITR expression levels in a panel of TNBC cell lines. (F-H) Western blot analyses of HDAC9 isoform knockout efficiency in MDA-MB-231, 231-PTX

and BT-549 cells. (I) Tumor growth curve with 10 ng/kg paclitaxel injected every 2 d or the same volume of normal saline (6 total injections). Tumor volumes were measured and compared using a two-sided Student's test (*: $p < 0.05$; **: $p < 0.01$). (J) Fluorescence imaging of HDAC9 knockout or control tumors after 6 cycles of PTX treatment. (K) Western blot of cyclin B1 in HDAC9 knockout and control BT-549 cells. (L) Western blot of acetylated α-tubulin in HDAC9 knockout and control BT-549 cells.

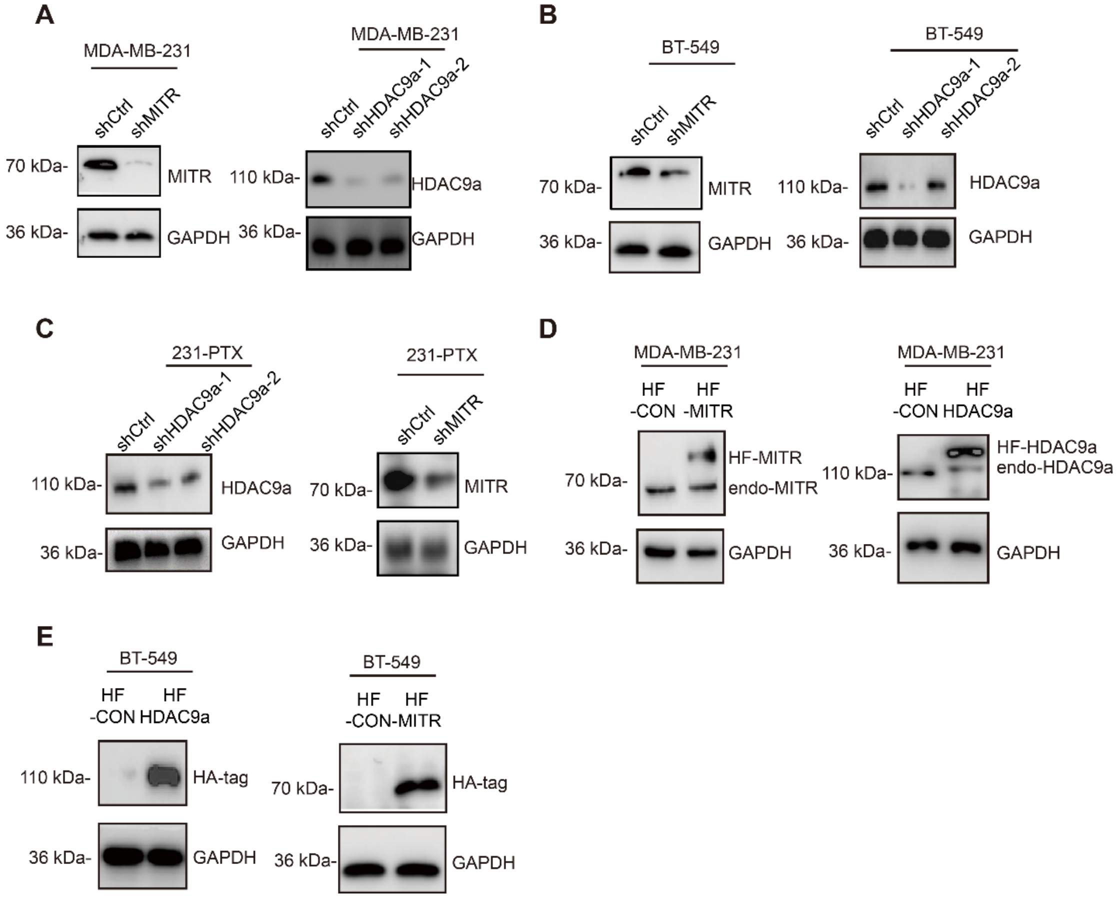

Figure S4: Similar phenotypes of MITR and HDAC9a in BT-549 cells. (A-C) Knockdown efficiency of MITR and HDAC9a in MDA-MB-231, 231-PTX and BT-549 cells. (D) Exogenous MITR and HDAC9a efficiency in MDA-MB-231 andBT-549 cells.

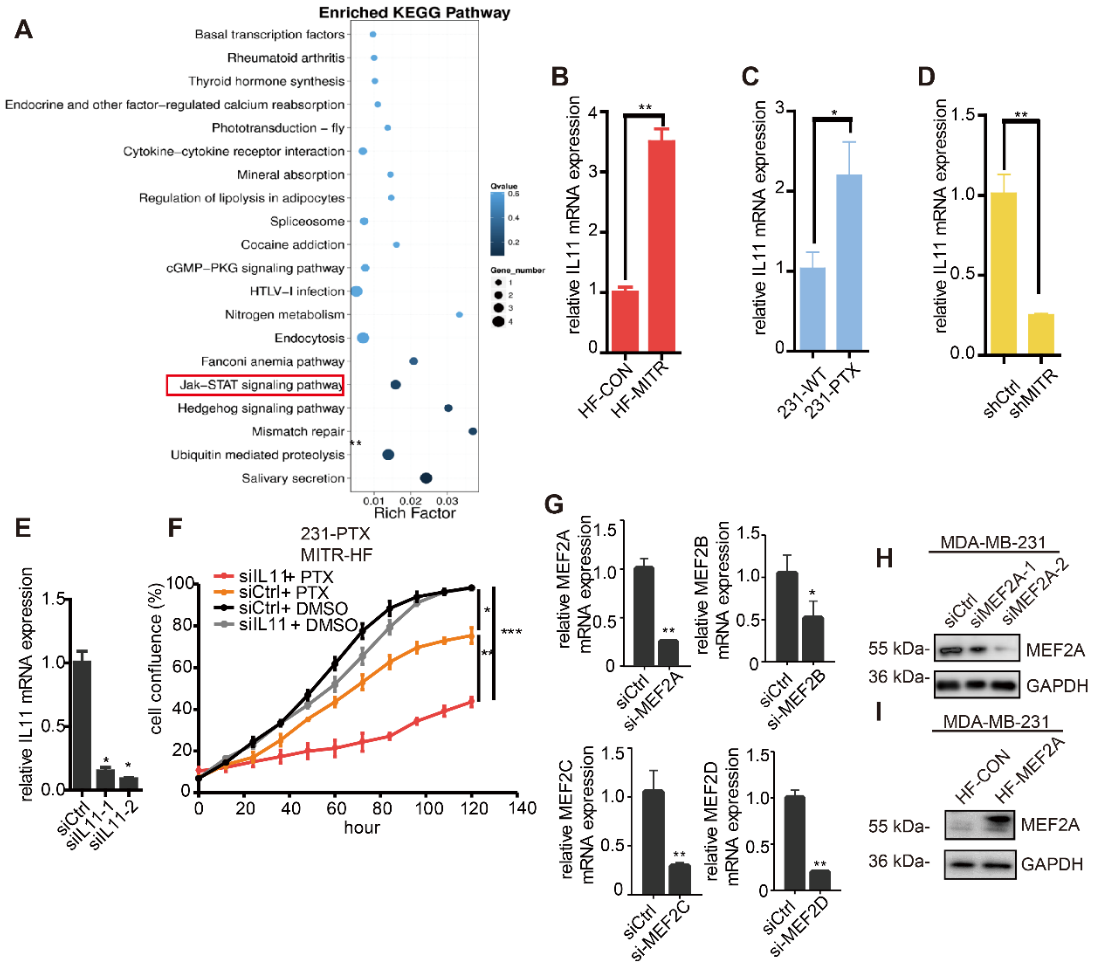

Figure S5: IL11 and MEF2A expression levels. (A) Top 20 enriched KEGG pathways comparing RNA sequencing of shMITR to shCtrl. (B-D) IL11 mRNA quantification by qPCR in cells stably expressing MITR, stable knockdown of MITR and with paclitaxel resistance (*: $p < 0.05$, **: $p < 0.01$). Data were expressed as the mean ± s.e.m. in three independent experiments. (E) IL11 silencing efficiency of two siRNA binding IL11 (*: $p < 0.05$). (F) 231-PTX MITR overexpression cells were silenced by siIL11 and were cultured in media containing 100 nM PTX for 140 h. Cell confluence was measured and compared using a two-sided Student's test (*: p value < 0.05; **: p value < 0.01). (G) IL11 silencing efficiency of MEF2 family members with one siRNA for each gene. (H-I) Western blot of the silencing and overexpression of MEF2A in MDA-MB-231 cells.

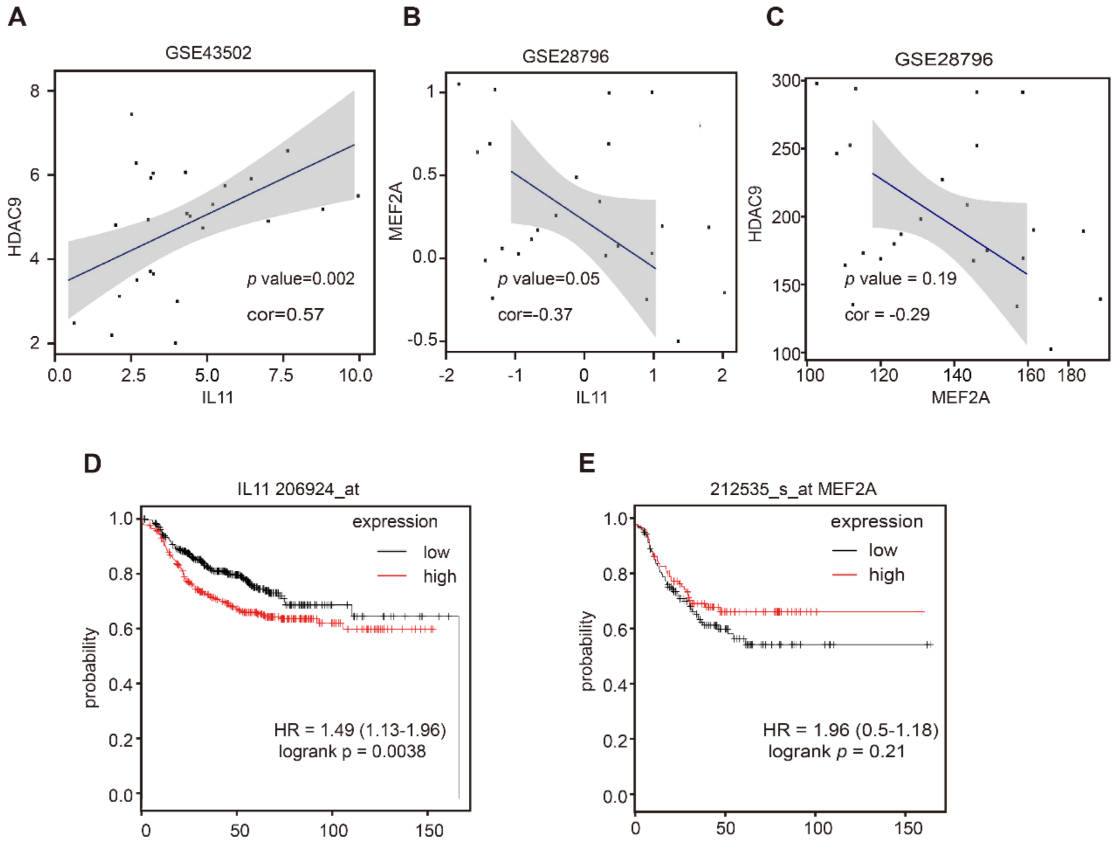

Figure S6. HDAC9, MEF2A and IL11 in public datasets. (A-C) Scatter plots of the correlation among HDAC9, MEF2A and IL11 mRNA expression in GEO public datasets. (A: t-test of $p < 0.05$, $R^2 = 0.57$ calculated by Pearson correlation, B: t-test of $p = 0.05$, $R^2 = 0.37$ calculated by Pearson correlation, C: t-test of $p > 0.05$, $R^2 = 0.29$ calculated by Pearson correlation). (D-E) Survival outcomes for TNBC patients based on IL11 and MEF2A mRNA expression from the KM plotter databases (D: $p < 0.05$, E: $p > 0.05$).

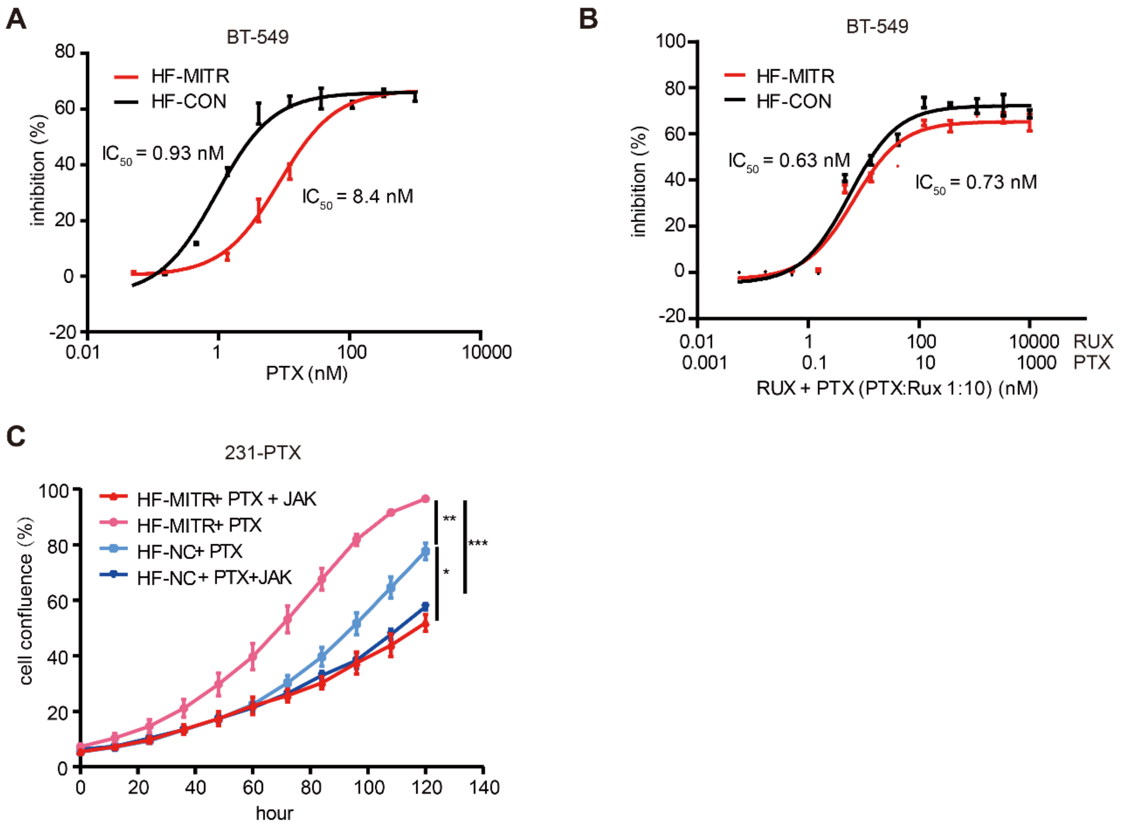

Figure S7. Combing therapy with JAK inhibitor has significance in 231-PTX and BT-549 cells. (A-B) Cell inhibition curve of HF-CON and HF-MITR in BT-549 cells treated with different doses of paclitaxel (A) or 1:10 ratio of paclitaxel (PTX) vs. ruxolitinib (RUX) (B) for 72 h. IC50 values of different groups were calculated and compared ($p < 0.05$ for A and $p > 0.05$ for B). (C) 231-PTX cells, transfected by HF-MITR and negative control were cultured in 10 nM PTX, 100 nM RUX, 10 nM PTX + 100 nM RUX and same amount of DMSO (*: $p < 0.05$; **: $p < 0.01$).

Supplementary Tables

Table S1. List of qRT-PCR primers.

Table S2: IHC clinical information.

Table S3. List of drug related pathways.

Table S4. RNA sequencing result of gene candidates

Table S5. CRISPR screening result of gene candidates.

Table S6. RNA sequencing result of HDAC family genes.
